# Coupled feedback regulation of nuclear factor of activated T-cells (NFAT) modulates activation-induced cell death of T cells

**DOI:** 10.1038/s41598-019-46592-z

**Published:** 2019-07-23

**Authors:** Sung-Young Shin, Min-Wook Kim, Kwang-Hyun Cho, Lan K. Nguyen

**Affiliations:** 1 Department of Biochemistry and Molecular Biology, School of Biomedical Sciences, Monash University, Clayton, Victoria 3800 Australia; 2 Biomedicine Discovery Institute, Monash University, Clayton, Victoria 3800 Australia; 3 Graduate School of Medical Science and Engineering, Korea Advanced Institute of Science and Technology (KAIST) 291 Daehak-ro, Yuseong-gu, Daejeon 34141 Republic of Korea; 4 Department of Bio and Brain Engineering, Korea Advanced Institute of Science and Technology (KAIST) 291 Daehak-ro, Yuseong-gu, Daejeon 34141 Republic of Korea

**Keywords:** Computational models, Systems biology

## Abstract

A properly functioning immune system is vital for an organism’s wellbeing. Immune tolerance is a critical feature of the immune system that allows immune cells to mount effective responses against exogenous pathogens such as viruses and bacteria, while preventing attack to self-tissues. Activation-induced cell death (AICD) in T lymphocytes, in which repeated stimulations of the T-cell receptor (TCR) lead to activation and then apoptosis of T cells, is a major mechanism for T cell homeostasis and helps maintain peripheral immune tolerance. Defects in AICD can lead to development of autoimmune diseases. Despite its importance, the regulatory mechanisms that underlie AICD remain poorly understood, particularly at an integrative network level. Here, we develop a dynamic multi-pathway model of the integrated TCR signalling network and perform model-based analysis to characterize the network-level properties of AICD. Model simulation and analysis show that amplified activation of the transcriptional factor NFAT in response to repeated TCR stimulations, a phenomenon central to AICD, is tightly modulated by a coupled positive-negative feedback mechanism. NFAT amplification is predominantly enabled by a positive feedback self-regulated by NFAT, while opposed by a NFAT-induced negative feedback via Carabin. Furthermore, model analysis predicts an optimal therapeutic window for drugs that help minimize proliferation while maximize AICD of T cells. Overall, our study provides a comprehensive mathematical model of TCR signalling and model-based analysis offers new network-level insights into the regulation of activation-induced cell death in T cells.

## Introduction

**Figure 1:**
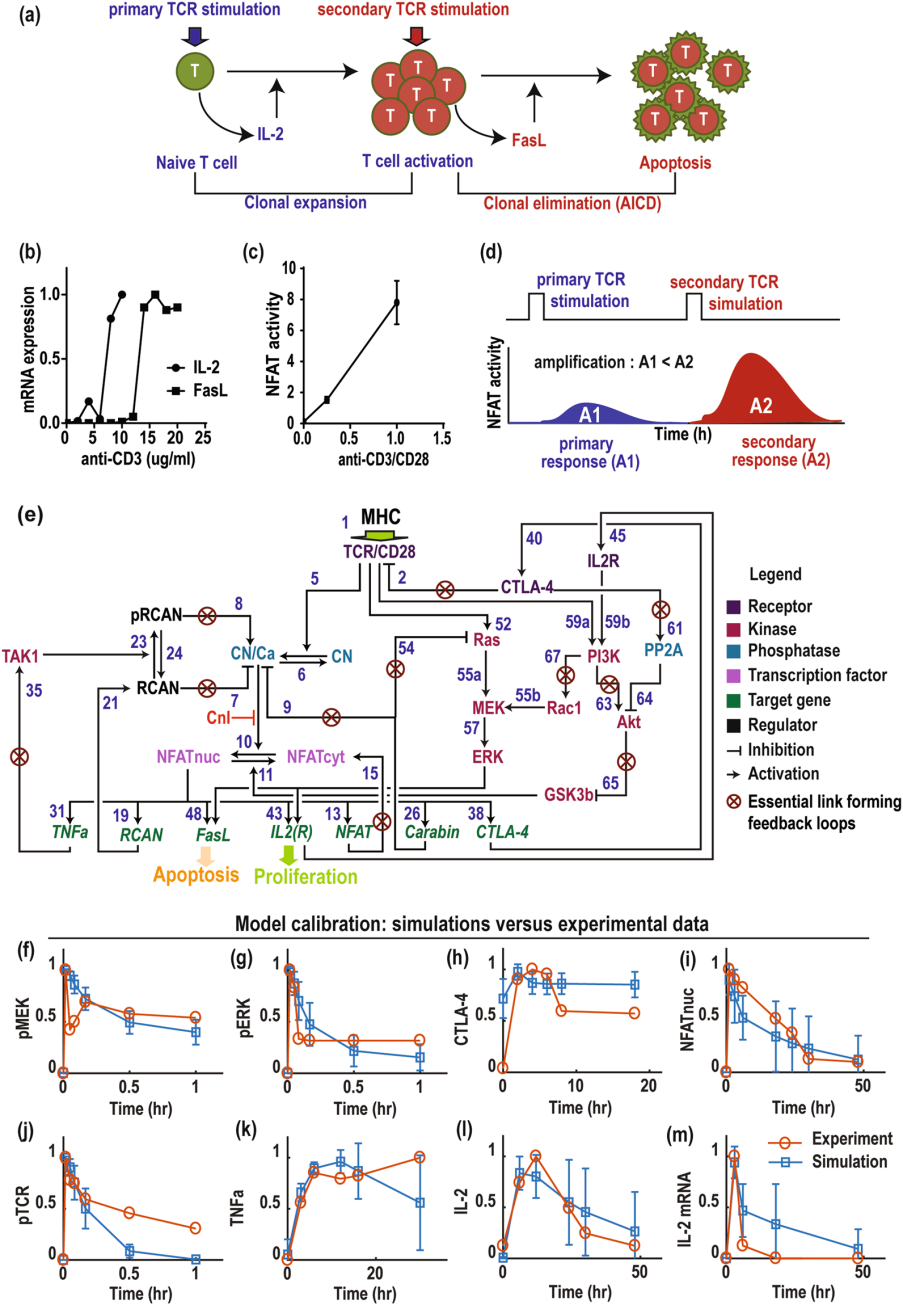
Mathematical modeling of the TCR-CN-NFAT signaling network. (**a**) An illustration of steps leading to AICD: the primary TCR stimulation promotes naïve T cell activation and clonal expansion through induction of IL-2; and the secondary stimulation (re-stimulation) triggers AICD through FasL induction and clonal elimination. **(b)** FasL requires ~2-fold stronger TCR stimulation (i.e. by anti-CD3) for transcription than IL-2. Data was reproduced from^6^. **(c)** A linear correlation between NFAT activity and anti-CD3/CD28 stimulation (reproduced from the^9^). **(d)** Graphical illustration of amplification of NFAT activity in response to sequential TCR stimulations. **(e)** A schematic reaction diagram of the TCR-CN-NFAT signaling network. Notations: CN/Ca, calcium bound active calcineurin; CN, inactive calcineurin; CnI, calcineurin inhibitor; NFATcyt, cytoplasmic (phosphorylated) inactive NFAT; NFATnuc, nuclear (dephosphorylated) active NFAT; pRCAN, phosphorylated RCAN at Ser 94 and 136, TNFa, TNFalpha, GSK3b, GSK3beta. **(f**–**m)** Model fitting to experimental data. Comparison of simulated (blue lines, showing the best-fitting model) and experimentally observed (red lines) time-courses. The time courses of pMEK **(f)**, pERK **(g)** and pTCR **(j)** were reproduced from the previous experimental data^47^. The time course of NFAT **(i)**, IL-2 **(l)**, IL-2 mRNA **(m)** were reproduced from the previous experimental data^49^. The time course of TNFa **(k)** were reproduced from the previous experimental data^48^. Error bars represent standard deviations, n = 100.

T lymphocytes (or T cells) are among the most abundant and versatile types of immune cells that protect human body against viral and bacterial infection^1,2^. Upon encountering an antigen, T cells proliferate and differentiate into specific effector cells such as cytotoxic T cells via a process called ‘clonal expansion’^1^ (Fig. 1a). However, due to the high proliferative capacity and the possibility of attacking the body’s own cells and tissues, the lifespan of antigen-activated lymphocytes must be effectively controlled to maintain T-cell homeostasis^2^. Activation-induced cell death (AICD) is a major molecular mechanism that eliminates activated T cells through apoptosis or cell suicide, as part of a process often referred to as ‘clonal elimination’^1^. Depicted in Fig. 1a, following an initial (primary) stimulation triggered by an antigen, AICD typically ensues as a result of a secondary activation (re-stimulation) of the T cell receptor (TCR) that is brought about by the persistence of the antigen hours/days after the first stimulation^2,3^. AICD occurs through interactions of death-inducing receptors and ligands, of which Fas and its ligand FasL are best characterized^3^, the latter is highly up-regulated upon TCR re-stimulation^4^.

The calcineurin-nuclear factor of activated T cells (CN-NFAT) signaling pathway downstream of TCR plays a central role in mediating an immune response to pathogenic stimulation^5^. Antigen-induced TCR stimulation elevates intracellular Ca^2+^ and subsequently activates the Ca^2+^-calmodulin-dependent serine/threonine phosphatase CN^5,6^. In turn, CN dephosphorylates NFAT, translocating it to the nucleus to transcribe target genes^5^. While the clonal expansion of T cells is driven mainly by induction of cytokines such as interleukin 2 (IL-2)^7^, the restimulation of T cells rapidly induces FasL instead^8^ (Fig. 1a), both are transcriptional targets of NFAT. This raised the question as to how T cells actively suppress FasL induction during the primary stimulation to avoid cell death, but potently induce it during the secondary restimulation. Using anti-CD3 as stimulus, Ryeom *et al*.^6^ found that FasL induction requires TCR stimulation ~2-fold stronger than that needed to induce IL-2 (Fig. 1b). Given anti-CD3 stimulation proportionately activates NFAT in T cells (Fig. 1c)^9^, these data suggest that the activity of NFAT should be highly amplified upon the secondary stimulation in order to effectively induce FasL expression, as illustrated in Fig. 1d. In line with this, specific NFAT isoforms were found highly expressed following a secondary stimulation^10–12^.

The activation of TCR is triggered by recognition of peptide antigens presented by major histocompatibility complex (MHC) molecules on antigen-presenting cells (APCs)^13^. Dendritic cells (DCs) are a major class of APCs implicated in the primary activation of naive T cells in the lymph nodes^14,15^ and are also the most potent antigen-presenting cells due to their high expression of MHC^14^. Following activation in lymph nodes, T cells migrate to target sites such as infected tissues and cells, where they encounter other types of APC including B cells and macrophages that trigger restimulation of TCR and promote AICD. Given the potent antigen-presenting power of DCs, the strength of the primary TCR stimulation is considered greater than that of the secondary one. This is supported by the observation that TCR undergoes endocytosis and degradation which renders it less sensitive to the secondary antigen stimulation^16^, and is further reinforced by the fact that TCR expression peaks during the clonal expansion phase and then decreases persistently^17,18^. How could a relatively weaker TCR stimulation trigger an amplified activation of NFAT during clonal elimination? This counterintuitive observation is puzzling and a mechanistic explanation remains obscure. Here, we aim to investigate the mechanisms underlying paradoxical NFAT amplification at a systems level. We test the hypothesis that this is an emergent feature arising from the complex structure and coupled feedback regulations within the signalling network downstream of TCR.

The ability to amplify input signals is critical and ubiquitous in many signal transduction pathways^19^. This ensures proper activation of downstream target genes even in response to suboptimal levels of the input. Signal amplification is also important in T cell signalling^20^. At the receptor level, co-activation of TCR by self-peptide–major histocompatibility complex (pMHC) and pMHC-independent transactivation was thought to amplify weak local stimuli from a few agonist pMHC molecules^20^. A ‘catch-and-release’ model for cyclical ZAP70 activation was also proposed as a potential mechanism for TCR signal amplification^21^. However, less is known about how signal is amplified downstream of TCR and at the transcriptional level, as in the case of NFAT. Various regulatory mechanisms are known to boost intracellular signalling, including the use of feedback loops^22,23^ and scaffold protein^24^. Given the complexity of TCR signalling, how NFAT amplification is achieved during AICD is poorly understood. It is also unclear whether such amplification is robust to molecular fluctuations.

To address these questions, we construct a new kinetic model of the integrated TCR/CN/NFAT-IL2/FasL signaling-transcriptional network (hereafter referred to as the TCR-CN-NFAT network) and analyze the system-level dynamic properties of this network. Our premise is that the nonlinear behaviors of such a complex network cannot be fully understood without formal quantitative modelling^25–31^. Our systems analysis demonstrates that the amplification of NFAT activation can be robustly achieved as a result of a coupled feedback structure. We identify the feedback mechanisms that have predominant control over NFAT amplification. Further, model simulations predict an optimal therapeutic window for a common class of TCR-targeted immunosuppressant agent, calcineurin inhibitor (CnI). Overall, our study provides novel network-level understanding of TCR signalling and the mechanisms underlying AICD in T cells.

## Results

### A mathematical model of the integrated TCR-CN-NFAT signalling network

To address if and how NFAT activation is amplified in response to weakening TCR stimulations, we constructed a comprehensive kinetic model of the signalling network downstream of TCR and calibrated this model against experimental data. The network interaction diagram (Fig. 1e) reconstructed from literature displays complex interlinked positive and negative feedback loops, encompassing multiple pathways including the CN-NFAT, PI3K/Akt and Ras/ERK MAPK pathways. The model was formulated by ordinary differential equations (ODEs) using a mixture of kinetic laws depending on the type of reactions (e.g. Michaelis-Menten kinetics for enzyme-catalyzed reactions, Hill kinetics for transcription, and mass-action kinetics for association/dissociation events)^26,27,32^. For full description of the model reactions, equations and parameter values, please refer to the Supplementary Information and Supplementary Tables S1–2. MHC serves as a key model input that triggers TCR activation; while IL-2 and FasL represent major outputs of the model that drive T cell proliferation and apoptosis (AICD), respectively^33,34^. Model implementation and all simulations were performed in MATLAB (The MathWorks. Inc. 2017b). An SBML version of this model is also available for download. Below, we summarize the salient regulatory mechanisms based on which the model was built, with an emphasis on various feedback loops. A more detailed description of these regulations is given in the SI.

#### Activation of T cell receptor and the TCR-CN-NFAT pathway

The activation of the T cell receptor is initiated by the recognition of cognate peptide–major histocompatibility complex (MHC) molecules on antigen presenting cells (APCs)^35^ (reaction 1, Fig. 1e), which lead to activation of the CN-NFAT, ERK and PI3K pathways (reactions 1, 5, 52 and 59a). Upon TCR activation, CN dephosphorylates multiple phosphoserine sites in the regulatory domain of NFAT, causing NFAT to translocate to the nucleus (reaction 10) and initiate specific transcriptional programs^36^.

#### RCAN-mediated negative and positive feedback loops

When unphosphorylated, RCAN inhibits CN through direct binding (reaction 7)^37,38^ but TAK1-induced phosphorylations of RCAN1 at Ser94 and Ser136 switch its role from being a CN inhibitor to an activator (reaction 8)^39^. As a result, RCAN mediates coupled positive and negative feedback loops towards NFAT^40^, described by reactions 19, 21 and 23.

#### IL-2 mediated positive feedback loop

TCR stimulation and CN-induced NFAT activation lead to transcription of the IL-2 receptor (IL-2R) and secretion of IL-2 (reaction 43). Secreted IL-2 activates IL-2R (reaction 45), triggering activation of the PI3K/Akt pathway. This mechanism consequently forms a positive feedback loop between IL-2/IL-2R, PI3K/Akt/GSK3 and NFAT.

#### NFAT auto-regulatory positive feedback loop

NFAT2/3 expressions are strongly induced following TCR stimulation and maintained by positive auto-regulation^12,41^. NFAT1/4 also form an auto-amplification feedback loop mediated through miRNAs^42,43^.

#### Carabin-mediated negative feedback loops

The gene Carabin has multiple consensus NFAT-binding sites and its expression is thus regulated by the CN signaling pathway^44^, forming a negative feedback loop (reactions 9 and 26). Moreover, Carabin suppresses RAS/ERK signalling following TCR activation^44^, which constitutes an additional negative feedback mechanism through Ras, described by reactions 26 and 54 (Fig. 1e).

#### CTLA-4-mediated negative feedback loop

The gene CTLA-4 has a consensus NFAT-binding sequence and binds NFAT with high affinity^45^ (reaction 2). Moreover, CTLA-4 is known to inhibit PI3K/Akt signalling by enhancing PP2A-induced dephosphorylation of Akt^46^ (reaction 61). Together, CTLA-4 mediates a negative feedback loop towards the TCR-CN-NFAT axis (reactions 38, 40, 2).

### Model calibration and parameter estimation

A model’s adequacy and predictive capacity are typically warranted by its ability to recapitulate experimental data, achieved through a process known as model training (or model calibration) where the model is fitted with the data. To this end, we fitted our model to time-resolved measurements of various network components in response to TCR stimulation in T cells by anti-CD3^44,47–49^, displayed in Fig. 1f–m. These include phosphorylated TCR, MEK, ERK; expression (protein and mRNA) levels of TNFα, IL-2, CTLA-4; and NFAT activity. While phosphorylated TCR, MEK and ERK responded very rapidly (peaking at 5 min) and transiently to the stimulation (Fig. 1f,g,j), the transcriptional responses such as that of IL-2, TNFα and CTLA-4 were much slower (Fig. 1i,k,l,m). Moreover, unlike IL-2’s transient induction, CTLA-4 and TNFα displayed a sustained expression pattern (Fig. 1h,k).

The previous experimental evidences demonstrated that NFAT activation is amplified by successive TCR stimulations^11^. Furthermore, FasL expression is suppressed following the primary activation but highly induced by the secondary one^6,50^. To recapitulate these qualitative features, we further calibrated the model by assuming that the level of NFAT abundance increased by at least 2 folds^51^ and FasL increased by at least 10 folds upon the secondary stimulation^6^. These fold change are based on previous observations^6^ suggesting that to properly induce ‘sufficient’ FasL to trigger T cell apoptosis upon secondary TCR stimulation, NFAT level should increase at least 2 folds compared to the primary stimulation. Moreover, although a 2-fold change in expression is often considered biologically meaningful, assuming a 10-fold increase for FasL provides a tighter constraint ensuring that it is sufficiently induced to activate apoptosis. The primary and secondary stimulations were modelled by sequential equally-strong pulsatile stimulations of TCR as depicted in Fig. 1d.

Model fitting was implemented using a genetic algorithm (GA) as part of the Optimization Toolbox in MATLAB to find the best fitted model parameter values (Table S3) that minimizes an objective function defined to measure the discrepancy between model simulations and data (see Material & Method and Supplementary Section S1 for a detailed description). Shown in Fig. 1f–m, model simulations using the best fitted set are highly concordant with experimental data (blue squares versus red circles), and reproduced the differential dynamic patterns and timescales of the network nodes. To evaluate how robust the best-fitted model is to parameter variation, we performed simulations (n = 100) where the fitted parameters were randomly varied within 30% of the fitted values. Importantly, the experimental data points largely reside within the variation ranges of the simulated curves (indicated by the blue error bars, Fig. 1f–m), indicating that the best-fitted model is robust in accounting for potential experimental noise.

### Network-level responses to sequential TCR stimulations

Accumulated experimental evidence have suggested that NFAT activation is paradoxically amplified in response to sequentially weakened TCR stimulations^6,11^. Here, we exploit our calibrated model to investigate this phenomenon. First, we performed dynamic simulations of all the network components in response to sequential and equally-strong pulsatile stimulations of TCR, which mimic the primary and secondary antigen-induced stimulations in T cells upon a pathogenic insult^14^. The time interval between the primary and secondary TCR stimulations was set to 48 hours, taking into account the minimal time required for activated T cells to migrate to the infected sites^52^.

**Figure 2:**
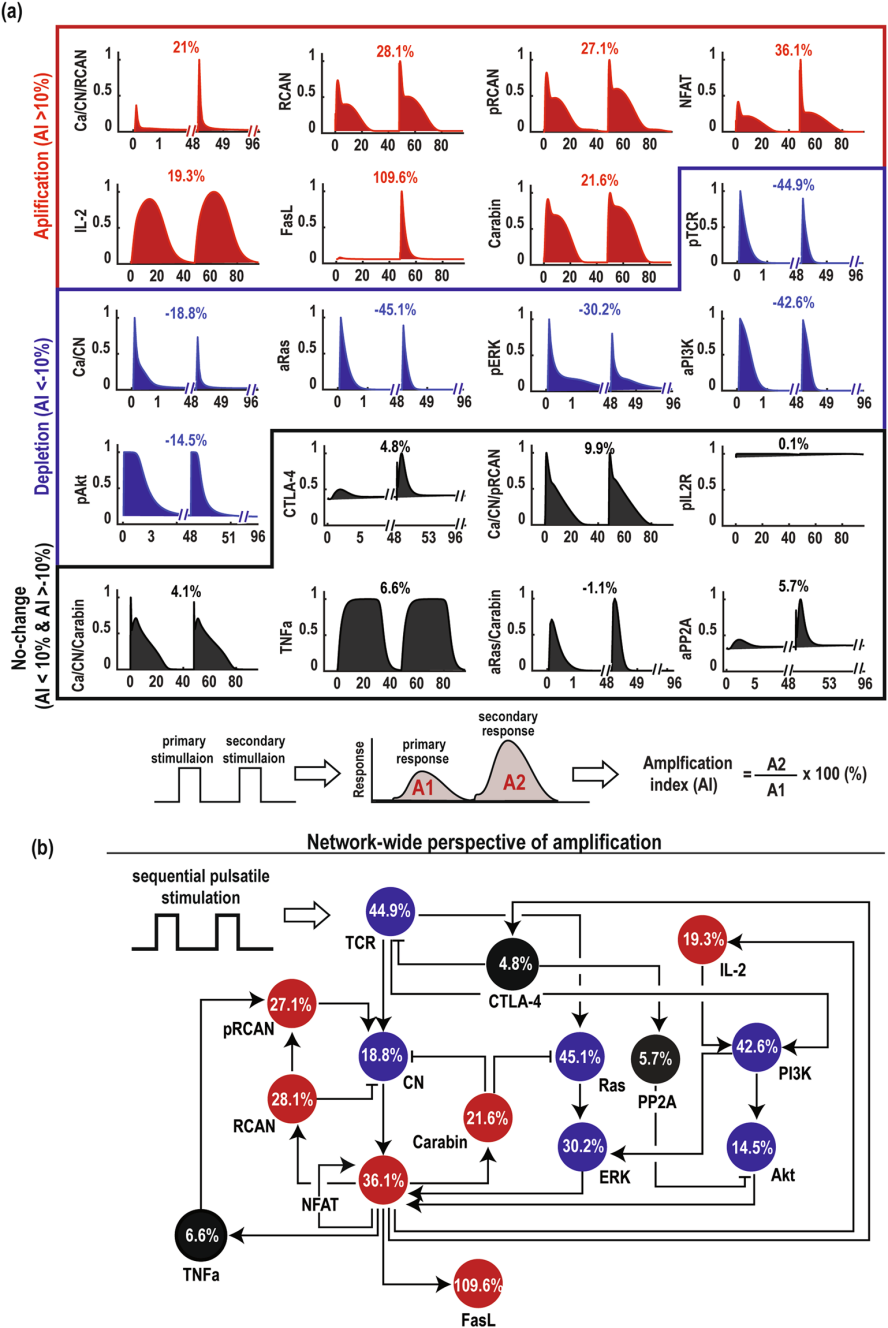
Network-level responses to sequential TCR stimulations. (**a**) Sequential TCR stimulations induced amplified responses for some signaling components (red) but depleted responses for others (blue). Black indicates no changes. The amplification index (AI) was defined as the fold-change (%) of the area under curve of the secondary response (A2) to that of the primary one (A1). **(b)** Responses of network components shown in (a) are mapped onto a simplified network.

Indeed, model simulations showed that with sequential TCR stimulations of equal strengths, the secondary stimulation provoked a strong amplification of NFAT activity, accompanied by a significant induction of FasL expression (Fig. 2a). However, not all the network components responded in an amplifying manner. In fact, as shown in Fig. 2a we could categorize the responses into three groups: (i) *amplification* (signified by an Amplification Index (AI) ≥ 10%, here AI is quantified as the percentage change of the area under curve (AUC) of the secondary response as compared to the primary one); (ii) *depletion* (i.e. AI < −10%); and (iii) *no-change* (i.e. 10% ≥ AI ≥ −10%). Due to the abrupt and highly transient responses observed for some network components, we use AUC instead of the maximal magnitude to quantify the amplification index as AUC better measures the dynamic ‘flux’ of signalling readouts^32,53^. Network components belonging to the *amplification* group include NFAT, FasL, CN/RCAN, RCAN, pRCAN, IL-2 and Carabin, (highlighted in red in Fig. 2a), of which NFAT and FasL display highest amplification (Fig. 2b). pTCR, Ca/CN, aRas, pERK, aPI3K and pAkt on the other hand, belong to the *depletion* group (blue, Fig. 2a) while the remaining nodes such as CTLA-4, CN/pRCAN, pIL2R, CN/Carabin, TNFa, aRas/Carabin and aPP2A did not show any significant changes (black, Fig. 2a). Importantly, the network response including amplification of the network components are robust to variation in the duration of the stimulation pulses (Figs S1–2).

Interestingly, members of each group are not necessarily clustered within the same signaling modules but instead scattered among the network (Fig. 2b), indicating signals do not simply propagate linearly but flow in a nonlinear manner. While the amplification of NFAT’s target genes (e.g. FasL, IL-2, RCAN and Carabin) can be intuitively attributed to the amplification of NFAT, and the depletion of Ras/ERK activities can be explained by the depleted activation of TCR and Carabin inhibition, explanation for other results, e.g. NFAT amplification or depleted PI3K/Akt signalling, are less straightforward. In these cases, there appear a competition between the positively- and negatively-effecting upstream elements but it is unclear just from the visual inspection, which one is prevailing. Together, these findings confirm the network’s ability to amplify NFAT activation in response to non-amplifying sequential TCR stimulations, and further highlight that predicting network response based on mere visual inspection or conventional way of pathway classification is insufficient, arguing for a more systematic approach.

### Intricate regulation of NFAT amplification and FasL induction by feedback mechanisms

**Figure 3:**
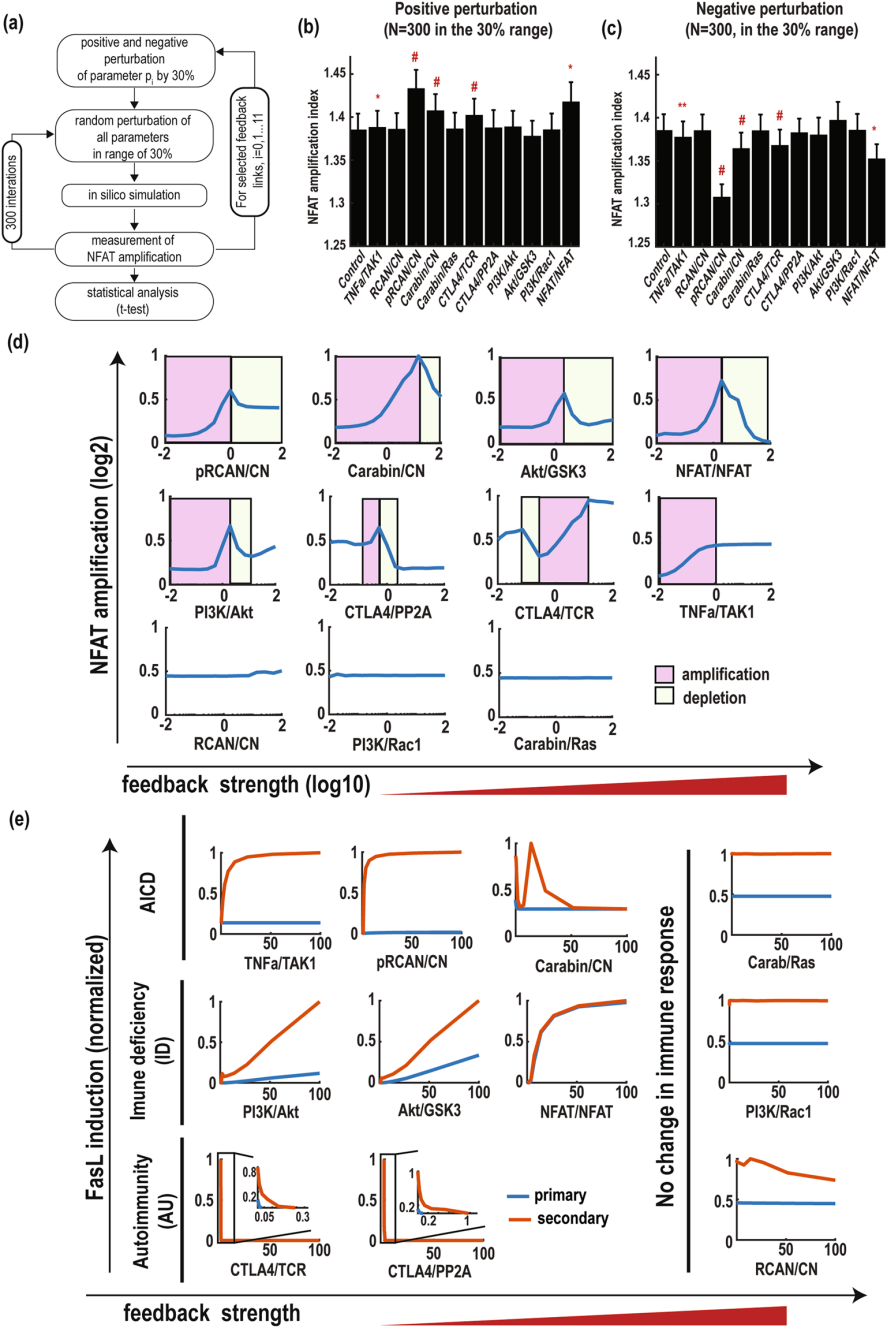
Perturbation analysis of the TCR-CN-NFAT network. (**a**) Workflow of the perturbation analysis. For each parameter, its nominal (i.e. best-fitted) value is firstly either positively (or negatively) perturbed by 30%, and then all model parameters are further randomly perturbed within 30% range of their nominal values. The parameter perturbation effect is compared with control condition (i.e. no perturbation of the target parameter). **(b**,**c**) Perturbation results obtained by positive **(b)** and negative perturbation **(c)** of the various feedback links, here ^#^indicates p < 0.001; **indicates p < 0.01; *indicates p < 0.05. **(d)** NFAT amplification patterns in response to gradual increase of feedback strength. **(e)** Classification of feedback loops according to the predefined immune response (AICD, immune deficiency and autoimmunity). Sequential TCR stimulations were applied as an input. The primary (blue lines) and secondary FasL responses (red lines) were obtained as output in response to a gradual increase of feedback strength.

The TCR-CN-NFAT signalling network contains multiple feedback loops that are highly interconnected and thus hamper an intuition-based analysis of the underlying mechanism of NFAT amplification. To examine which feedback mechanism(s) may contribute to such mechanism, we performed model-based feedback perturbation analysis. To this end, we systematically perturbed the molecular links (a total of 11 links denoted by red crossed circles in Fig. 1e, and listed in Supplementary Table 4) that form the key feedback loops by altering the kinetic parameters associated with these links (increasing/decreasing by 30% of the nominal values, see Fig. 3a for the workflow) and assessed the effect of these perturbations on NFAT amplification. To further test if the feedbacks’ effects may be influenced by other model parameters we repeated these simulations hundreds of times (n = 300) by randomly sampling all the remaining kinetic parameters within wide ranges, and the effect of each feedback loop was statistically compared with control scenarios (i.e. no perturbation). These simulations revealed that the feedback links pRCAN/CN, Carabin/CN, CTLA4/TCR and NFAT/NFAT displayed the most significant controlling effects on NFAT amplification, consistent for both positive and negative perturbations (Fig. 3b,c).

To further analyze how each of the 11 considered molecular links (or feedback mechanisms) regulate NFAT amplification, we simulated NFAT amplification response to a gradual increase in the strength of the feedback mechanism over very wide ranges (×10^−2^ to ×10^2^ of nominal strengths), as shown in Fig. 3d. Although various response patterns were displayed, three groups were identified: (i) monotonic increase (TNFa/TAK1), (ii) no change (RCAN/CN, Carabin/Ras, PI3K/Rac1) and (iii) biphasic (pRCAN/CN, Carabin/CN, CTLA4/PP2A, PI3K/Akt, Akt/GSK3 and NFAT/NFAT, including a reverse biphasic, CTLA4/TCR) responses. A biphasic response pattern is defined by an increase in the output at low level of the increasing input accompanied by a decrease in the output at high level of the input; while a monotonic increase (decrease) pattern is defined by a monotonic change in the output against an increasing input. Interestingly, the four dominant mechanisms identified earlier all belong to the biphasic (or reverse biphasic) group, suggesting they have dual roles in controlling NFAT amplification. For example, an initial reinforcement of the NFAT/NFAT autoregulatory loop promotes NFAT amplification but further strengthening switched to suppress amplification instead (Fig. 3d). These simulations indicate that the dual-functional feedbacks are more dominant in controlling NFAT amplification. Furthermore, as a consequence of the biphasic dependency, NFAT amplification is most pronounced within a confined range of the feedback strengths. Importantly, we found that 4 feedback mechanisms (pRCAN/CN, PI3K/Akt, Akt/GSK3 and NFAT/N/NFAT) of the biphasic group displayed highest NFAT amplification at (or around) their nominal strengths (fitted parameter values), suggesting that the network is endowed with an ability to amplify NFAT activity upon secondary stimulations under normal physiological contexts.

FasL is a target gene of NFAT and involved in T cell apoptosis during an immune response^54^. Thus, FasL should be initially silenced to ensure normal T cell activation and proliferation, as otherwise it would lead to early cell death and immune deficiency^6^. In contrast, if there is no induction of FasL by the secondary TCR stimulation, which mainly occurs at the infection site, this may lead to autoimmunity due to increased cytotoxic T cells^55^. To further classify the functional role of the above feedback mechanisms in immune disease contexts, we defined three types of immune response according to the simulated patterns of FasL induction: AICD, Immune Deficiency (ID) and Autoimmunity (AU) (Fig. 3e). Specifically, AICD is characterized by silenced FasL by the primary stimulation coupled with highly induced FasL by the secondary stimulation; ID is characterized by a strong primary induction of FasL regardless of the secondary response; and AU is featured by silence of FasL induction to both the primary and secondary stimulations.

As shown in Fig. 3e, simulations of FasL expression predict that the feedback links TNFa/TAK and pRCAN/CN significantly increased the primary FasL induction but did not affect the secondary response. This suggests these feedback loops may be important in maintaining AICD and thus were functionally grouped into the AICD type. On the other hand, the links PI3K/Akt, Akt/GSK3, and NFAT/NFAT significantly promoted primary FasL induction, and were thus grouped into the ID type. The links CTLA4/TCR and CTLA4/PP2A suppressed both the primary and secondary FasL induction, and were therefore grouped into the AU type. The remaining feedback links including Carabin/Ras, PI3K/Rac1 and RCAN/CN did not show significant change in FasL induction (Fig. 3e, right). Note that Carabin/CN increased the secondary FasL induction at a lower strength but decreased it at a higher strength, and so can be grouped to AICD or AI types depending on its strength. Together, these computational analyses suggest that by differentially regulating FasL induction profile, different sets of feedback mechanisms underlie different biological phenomena including immune-related conditions such as ID or AU.

### NFAT amplification is robust to changes in TCR stimulation intensity

**Figure 4:**
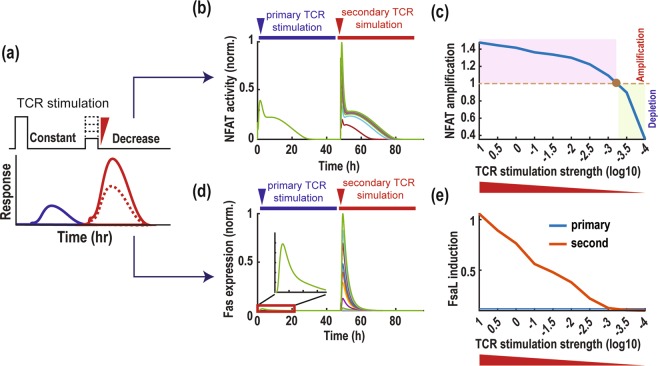
Robustness of NFAT amplification. (**a**) Illustration of sequential TCR stimulation pulses where the amplitude of the secondary pulse is gradually decreased at a fixed strength of the primary pulse. NFAT activation profile is taken as the model readout. **(b)** Time profiles of NFAT activation and **(c)** NFAT amplification index (AI) in response to varying TCR simulation strength. NFAT AI was measured as the ratio of the AUC of the secondary response to the primary one. The time profiles of FasL **(d)** and its induction primary/secondary ratio **(e)** in response to varying TCR simulation strength.

Dendritic cells (DCs) have been reported to have the most potent antigen presenting power and involved in the activation of naive T cells^15^. Other professional APCs that have relatively lower antigen presenting power, including macrophages and B-cells, are predominantly populated at the infection sites where activated T cells are re-stimulated^56^. These biological observations led us to hypothesize that even a relatively much weaker secondary TCR stimulation would be able to provoke NFAT amplification. To test this hypothesis, we performed simulations by subjecting the model to sequential TCR stimulations where the secondary stimulation was increasingly weakened compared to a fixed primary stimulation (Fig. 4a). Interestingly, model simulations showed that NFAT activity still displayed robust amplification in response to a significantly reduced secondary stimulation (Fig. 4b). Surprisingly, NFAT amplification was maintained even when the secondary TCR stimulation was reduced by 1000 folds of the nominal strength (Fig. 4c). Consistently, FasL expression was also induced at high levels by a much weakened secondary stimulation (Fig. 4d,e).

Taken together, these analyses reveal that the TCR signalling network is strongly robust in enabling NFAT amplification and FasL induction, and capable of doing so under situations where the secondary TCR stimulation is comparatively weaker than the primary one, which is often the case in AICD.

### Identification of optimal therapeutic window maximizing the effect of calcineurin inhibition

Cyclosporin A (CsA) is an immunosuppressant drug widely used to prevent graft rejection in organ and tissue transplantation through inhibition of IL-2 expression and T cell proliferation^57,58^. CsA is known to bind to the catalytic domain of calcineurin and inhibits its phosphatase activity^59,60^. However, once graft rejection reaction occurs, CsA treatment may lead to AICD suppression and thus potentiates immune rejection. Interestingly, conflicting experimental data have been reported on the effect of CsA. For instance, Kerstan *et al*.^61^ found that CsA promotes to Fas-mediated apoptosis (AICD) in rat lymph node T cells, whereas Shi *et al*. and Yazdanbakhsh *et al*.^62,63^ suggested that CsA blocked AICD in T cell hybridomas *in vitro* and in thymocytes *in vivo*. Moreover, Kadereit *et al*.^64^ reported that CsA did not affect AICD in either umbilical cord blood T cells or adult T cells. These observations led us to hypothesize that calcineurin inhibition may exert dual effect on AICD and such context-specific effect likely results from the complex network interactions. Here, we investigate this hypothesis using model simulations. To keep the analysis general, instead of referring to a specific CN inhibitor (e.g. CsA or Tacrolimus)^65^ we use CnI to indicate a generic inhibitor of CN.

**Figure 5:**
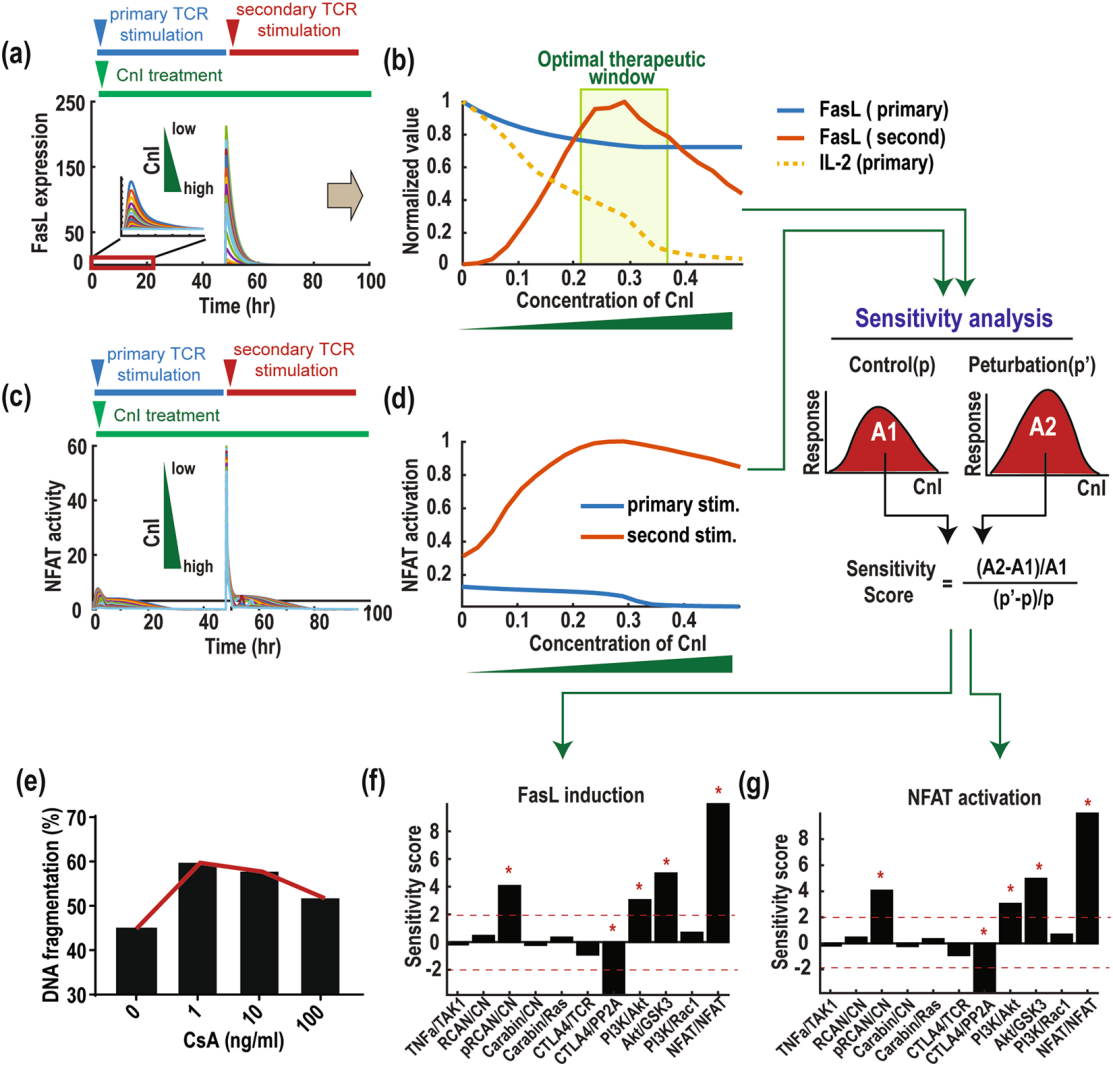
Optimal therapeutic window optimising CnI effect. (**a**) Effect of CnI treatment on FasL induction. **(b)** Biphasic response of the secondary FasL induction to a gradual increase of CnI. **(c)** Effect of CnI treatment on NFAT activation. **(d)** The response profiles of NFAT activation. **(e)** Biphasic response of DNA fragmentation to increasing CsA dosage (data was reproduced from^58^). **(f,g)** Sensitivity analysis of feedback mechanisms for the CnI effect on the secondary FasL **(f)** and NFAT **(g)** responses.

We performed time-dependent (Fig. 5a) and dose-response (Fig. 5b) simulations of FasL and IL-2 expression in response to increasing level of CnI treatment. Interestingly, while CnI monotonically suppress IL-2 and FasL’s primary response, it controls the secondary response of FasL in a biphasic manner (Fig. 5b). As the level of CnI is gradually raised, FasL initially increased, reached a peak and then decreased. This dose-dependent effect of CnI may contribute to the conflicting observations in previous studies of CnI’s role in T cells. Importantly, these simulation results indicate the existence of an optimal therapeutic window (range) for CnI within which, the agent would efficiently suppress IL-2 and at the same time potently induce FasL (highlighted in Fig. 5b), thereby effectively inhibiting the immune response.

As both FasL and IL-2 are target genes of NFAT, we further simulated the effect of CnI on the activation profile of NFAT (Fig. 5c,d). Interestingly, increasing CnI consistently suppress the primary activation of NFAT but affect its secondary activation in a biphasic manner (Fig. 5d). This indicates that CnI treatment may promote NFAT amplification as it facilitates the secondary but inhibits the primary NFAT activation. Further, our simulations suggest that CnI inhibits T cell proliferation through inhibition of IL-2, and promotes AICD through induction of FasL. These predictions are qualitatively in line with experimental data showing CN inhibitor using CsA indeed triggers a biphasic response in DNA fragmentation^58^, a hallmark of cell apoptosis (Fig. 5e).

Next, we sought to examine the functional effect of the various feedback loops on network response to CnI treatment using sensitivity analysis (see the Materials and Methods section for detail). For this, we perturbed each feedback mechanism and calculated the corresponding change in the area under curves (AUCs) of dose-response curves of FasL and NFAT (as in Fig. 5b,d). Note that the secondary responses were taken as readouts for the sensitivity analysis due to their importance in AICD. The sensitivity score quantifies the effect of the feedback mechanisms: positive scores indicate positive roles and vice versa (Fig. 5f,g). Notably, we found that the NFAT/NFAT self-regulated positive feedback exhibits the most significant impact on drug response, followed by the links pRCAN/CN, CTLA4/PP2A, PI3K/Akt, PI3K/GSK3. This finding is consistent with our earlier model prediction that the RCAN-, CTLA-4- and NFAT-mediated feedback mechanisms have significant regulatory effect on NFAT amplification (Fig. 3b,c).

### Coupled feedback regulation modulates NFAT amplification

**Figure 6:**
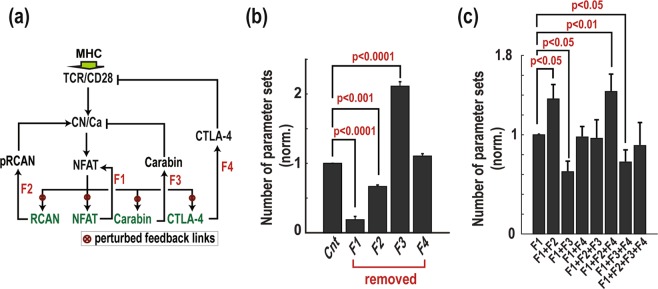
Coupled feedback regulation modulates NFAT amplification. (**a**) Schematic diagram of a reduced TCR-CN-NFAT model containing four key feedback loops. **(b)** Effect of feedback removal on NFAT amplification robustness. Cnt denotes nonperturbed model. **(c)** Effect of feedback addition on NFAT amplification robustness: the feedback loops are added to an initial model containing only F1.

The above results collectively indicate that four feedback loops, the NFAT auto-regulatory and pRCAN/CN, Carabin/CN, CTLA4/TCR loops, are most critical in controlling NFAT amplification. Here we seek to examine their specific role in either promoting or suppressing NFAT amplification and identify the core network structure underlying amplification. To this end, we developed a new reduced model of the TCR network that includes only the four loops, depicted in Fig. 6a (the model is described in detail in Supplementary Tables S5–6). To interrogate the role of individual feedback loop, we first removed each loop (i.e. F1-F4 in Fig. 6a) one by one from the model and assessed the effect of such removal on the robustness of NFAT amplification, indicated by the number of parameter sets triggering NFAT amplification when the remained model is randomly ‘sampled’ within the parameter space, as compared to the unperturbed model. Supplementary Fig. S5 displays amplified NFAT responses to sequential TCR stimulations in the unperturbed model, superimposed for 400 randomly sampled parameters sets. Removal of the positive self-regulatory feedback of NFAT (F1) significantly decreased NFAT amplification robustness, reducing the NFAT-amplifying parameter sets to <20% of the unperturbed network (Fig. 6b). Removing F2 also significantly reduced the NFAT-amplifying sets, but to a lesser extent. These data suggest F1 plays a strong promoting role towards NFAT amplification, which is followed by F2. In contrary, removing F3 significantly increased NFAT amplification robustness, indicating its strong inhibitory role; while removing F4 did not show any statistically significant effect on the robustness.

To further elucidate the contribution of each feedback loop to induction of NFAT amplification, we assessed its robustness when feedback mechanisms are incrementally added to the model containing just F1. We found that F1-only model is as robust as the full reduced model in enabling NFAT amplification (1^st^ vs. 8^th^ column, Fig. 6c), which indicates F1 alone is sufficient to induce amplification and is in line with its strong promoting role. Adding F2 or F3 either significantly enhanced or suppressed NFAT amplification, respectively (2^nd^ and 3^rd^ columns, Fig. 6c), consistent with their previously identified roles. The opposing roles of F2 and F3 is further evident as adding them together did not significantly change NFAT amplification robustness (5^th^ vs 1^st^ column) due to their effects being cancelled out. Further, adding F4 did not significantly change NFAT amplification robustness. Taken together, these analyses reinforced the coupled roles of the positive and negative feedback mechanisms in governing NFAT amplification, of which the self-regulatory positive feedback sufficiently and potently drives amplification that is strongly opposed by the Carabin/CN negative feedback loop.

### Ensemble modeling corroborate feedback functions in generalized network contexts

**Figure 7:**
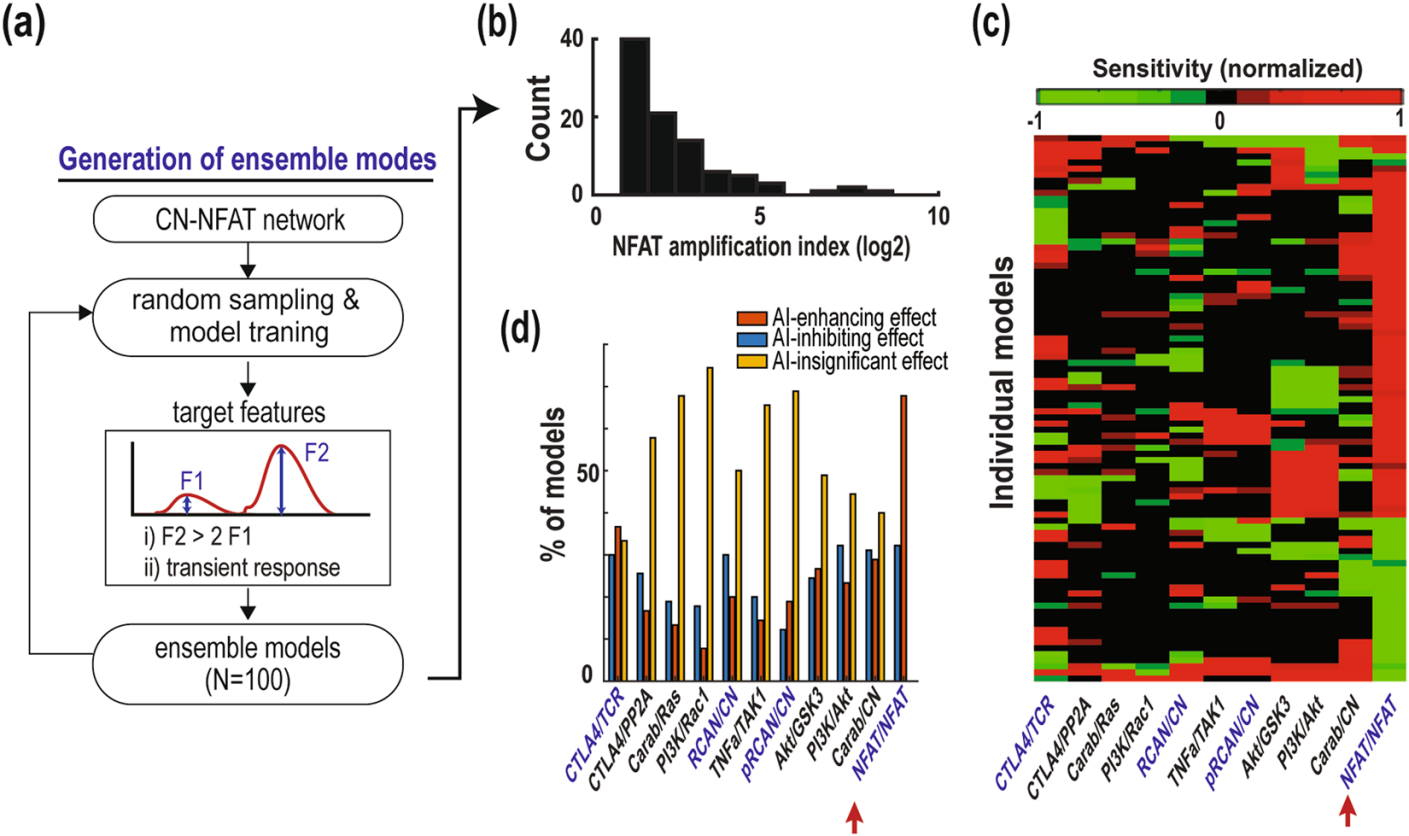
Ensemble modeling and simulations. (**a**) Flowchart of the ensemble modelling procecss. A random parameter set was selected for the initial values of the genetic algorithm (GA). Using GA, an individual model is calibrated to regenerate the target features. This process was repeated to obtain 100 ‘trained’ models. **(b)** Histogram distribution of the NFAT amplification index. **(c)** Heatmap showing the color-coded sensitivity scores from a sensitivity analysis of various model feedback mechanisms on NFAT amplification. The models were clustered using a hierarchical clustering method with Euclidean distance metric and average linkage. **(d)** Tabulation of individual models where each feedback displays an AI-enhancing (red) or AI-inhibiting effect (blue).

Using the best-fitted parameter set, we have shown in previous sections that the NFAT auto-regulatory and pRCAN/CN, Carabin/CN, CTLA4/TCR feedback loops play determining roles in inducing and regulating NFAT amplification. To see if this is also the case in more generalized cellular contexts, i.e. the model kinetic parameters are less constrained, we employed an ‘ensemble modelling’ strategy where multiple rather than a single optimal parameter sets are generated by constraining our model against a set of ‘desired’ qualitative features^66–69^ (see Materials and Methods). This process effectively generates an ensemble of models satisfying a common set of conditions, based on which simulations will be simultaneously performed and analyzed. Illustrated in Fig. 7a, we enforced two experimentally-observed constraints for the qualitative fitting: *(i)* the peak amplitude of the secondary NFAT activation response is significantly higher (≥2 folds) than that of the primary response^11^; and *(ii)* the NFAT activation response is transient in both cases^49^. The 2-fold cut-off was chosen to ensure that the fitted models display strong NFAT amplification. Moreover, transient response of NFAT was defined as having its level reaching a peak at ~1 hr following TCR stimulation which is subsequently reduced at late time points (>48 hr), reflecting the pattern seen in experimental data (Fig. 1i).

A total of 100 parameter sets and so individual models were generated from the fitting process, all reproduced the two constraints above, as shown by the model-specific (Supplementary Fig. S6) simulation of NFAT response curves. Figure 7b displays the histogram distribution of the NFAT amplification index (AI) across the individual models, with values ranging from 2 to 400 and median value of 3.4. To investigate the influence of feedback loops on NFAT amplification control in these models, we performed sensitivity analysis. In each model, each feedback link was suppressed by 50% (Materials and Methods) and the effect on NFAT amplification (AI) was assessed. A positive (negative) sensitive score indicates that the feedback enhances (inhibits) NFAT amplification (Fig. 7c). Consistent with our previous findings, clustering analysis of the sensitivity scores shows that the 4 feedback loops identified to be important for AI control (using the best-fitted parameters, section 2.7) are also among the most influential loops among the ensemble models (Fig. 7c). Next, to filter out the models where feedback inhibition has insignificant effect, we tallied only those in which the feedback exerts either a strong enhancing or inhibiting effect, i.e. sensitivity score within the 75^th^ or 25^th^ quantile, respectively. This was done for each of the 11 feedback loops. The results (Fig. 7d) reveals that the NFAT auto-regulated feedback strongly enhances NFAT amplification in ~70% of all models, followed by the CTLA4/TCR (enhancing-effect in 37% of the models), Carab/CN (29%) and PI3K/Akt (23%) feedbacks. Interestingly, the NFAT positive feedback is the only one where its perturbation always results in strong influence on AI. Consistent results were also obtained when we perturbed the feedback loops by increasing their strength instead (Supplementary Fig. S4), or varied the level of perturbation (Supplementary Fig. S3). Taken together, these results indicate that the NFAT auto-positive feedback plays a predominant role in promoting NFAT amplification, even in a generalized context. The role of the other feedbacks however are more context dependent.

## Discussion

Despite its significance, the mechanisms underlying signal amplification in response to sequential input stimulations in biological systems are not fully understood. This issue is acute in the context of activation-induced cell death of T cells, which amplifies the signaling response to successive TCR stimulations by a specific antigen^10,11^. Upon the primary stimulation, IL-2 is expressed which promotes T cell proliferation and clonal expansion. In contrast, the secondary stimulation triggers cell death through induction of FasL. Multiple lines of evidence proposed that the amplification of NFAT plays a critical role in orchestrating these processes^6^. In this study, we have employed systems-based modelling to analyse the dynamic properties of NFAT-driven AICD and identify the hidden mechanisms underlying these properties^25–27^. This was done through development of a new computational dynamic model of an integrated multi-pathway TCR signalling network, based on which we studied NFAT amplification in response to sequential TCR stimulations.

Our model simulations and analyses revealed that NFAT activity can be robustly amplified in response to non-amplifying TCR stimulations, and that such amplification is induced primarily by a coupled positive feedback structure comprising of an auto-regulatory positive feedback of NFAT itself, and a positive feedback loop from NFAT to CN/Ca mediated via phosphorylated RCAN. Simulations of reduced models showed that the former feedback loop is the major mechanism driving NFAT amplification, which is further enhanced by the presence of the latter feedback mechanism. Furthermore, model analysis demonstrated that the negative feedback loop regulated by Carabin towards CN/Ca is most critical in suppressing NFAT amplification. The mixed positive and negative feedback regulation within the TCR signaling network thus provides a previously unknown circuit design that robustly tunes NFAT amplification as T cells encounter successive antigen stimulations.

AICD is a major mechanism controlling clonal elimination of T cells during an immune response^1,2^, and plays a key role in eliminating the activated T cells during transplantation rejection response^58,70–73^. Cyclosporine A, a synthetic analogue of RCAN, is the most effective and widely used immune suppressive drug in transplantation immunology^74^. Our new model demonstrates the potential for therapeutic exploration through the investigation of CnI-induced network response. Model simulations demonstrated that CnI monotonically inhibits IL-2 expression but induces a biphasic response in FasL expression. This provides new insights into the network-level mechanism underlying CnI action in T cells. Importantly, our *in silico* analysis uncovered a potentially optimal therapeutic window for CnI. As T cell activation is regulated by complex processes and feedback mechanisms^75^, identifying such an optimal range for CnI concentration which minimizes T cell activation (proliferation) but maximizes T cell apoptosis has been a challenging task in clinical applications^58^. Our predicted CnI therapeutic window forms a clinically relevant hypothesis that awaits experimental validation in the future.

In summary, we have developed a novel integrative model of the TCR signalling network encompassing multiple pathways to investigate the phenomenon of NFAT amplification in AICD. Our computational analysis has provided deeper insights into the mechanistic understanding of T cell biology. We envision that the model could be further exploited in future research for exploring network-level effects of existing and/or novel drugs targeting the TCR network across different diseases.

## Materials and Methods

### Mathematical modelling and model calibration

The mathematical model was implemented in MATLAB (The MathWorks. Inc. 2017b), and the Global Optimization toolbox (The MathWorks. Inc. 2017b) was employed for model calibration and parameter estimation. Specifically, GA (genetic algorithm) was used to find the best kinetic parameter values that minimize discrepancy between the simulations and training data sets^76^. GA is a powerful method and widely used to find approximate solutions of optimization problems in the systems biology field^27,30,31,76^. GA is stochastic, powerful and effective methods to solve both constrained and unconstrained optimization problems based on a natural selection process that mimics biological evolution^77,78^. The algorithm repeatedly modifies a population of individual solutions. At each step, the genetic algorithm creates new individuals using crossover and mutation as well as selection of best individuals from the current population, and uses them as parents to produce the offspring for the next generation. Over successive generations, the population ‘evolves’ toward an optimal solution. Model simulations using the best-fitted parameter set showed that the model quantitatively recapitulates all the observed dynamics of the TCR-CN-NFAT network very well (Fig. 1f–m). For parameter estimation, high-performance super-computers at Monash University (http://www.monash.edu) were utilized, consisting of two Haswell CPU sockets with a total of 16 physical cores (or 32 hyperthreaded cores) at 3.20 GHz and 300 TB usable storage.

Kinetic parameters were constrained within biologically plausible ranges before fitting, informed by previous work of other groups^32,79^, and also ours^27,80,81^. For examples, ka (association rate) = [1e-4, 1e4] (nM^−1^ min^−1^); kd (dissociation rate) = [1e^−4^, 1e4] (min^−1^); kc (catalytic rate) = [1e-4, 1e5] (min^−1^); Vmax (max velocity) = [1e-4 1e4] (nM min^−1^) and Km (Michaelis-Menten constant) = [1e-4, 1e5] (nM).

### Sensitivity analysis of feedback loops

Parameter sensitivity analysis is the process of determining the sensitivity of responses to the change of parameter values^82^. It has been recognized as a powerful tool for systems biological approaches due to its practical applicability to model building and evaluation, understanding system dynamics, evaluating the confidence of a model under uncertainties, and experimental design^83–85^. In general, sensitivity functions for parameter sensitivity analysis take the form

$${S}_{P}^{M}=\frac{\partial M/M}{\partial P/P}=\frac{Percentage\,change\,in\,M}{Percentage\,change\,in\,P}$$

where *M* denotes the response of a system and *P* denotes a system parameter^86^. In this study, we employed a relative sensitivity function known as the logarithmic sensitivity function (LSF). LSF is dimensionless and thereby allows comparison of physically different parameters^87^. The LSF ${L}_{ji}$ is defined as follows:

$${L}_{ji}=\frac{\partial \,\mathrm{ln}\,{x}_{j}({p}_{i})}{\partial \,\mathrm{ln}\,{p}_{i}}=\frac{{p}_{i}}{{x}_{j}({p}_{i})}\cdot \frac{\partial {x}_{j}({p}_{i})}{\partial {p}_{i}}\approx \frac{{p}_{i}}{{x}_{j}({p}_{i})}\cdot \frac{{x}_{j}({p}_{i}+{\rm{\Delta }}{p}_{i})-{x}_{j}({p}_{i})}{{\rm{\Delta }}{p}_{i}}=\frac{\frac{{x}_{j}({p}_{i}+{\rm{\Delta }}{p}_{i})-{x}_{j}({p}_{i})}{{x}_{j}({p}_{i})}}{\frac{{\rm{\Delta }}{p}_{i}}{{p}_{i}}}$$

where ${x}_{j}$ denotes the *j*_th_ state variable (output of interest), *p*_*i*_ the *i*_th_ parameter, and ${\rm{\Delta }}{p}_{i}$ the change of the parameter *p*_*i*_.

### Ensemble modelling

For ensemble modelling, we first randomly selected a set of kinetic parameter value in the range of −4 to 4 on a logarithmic scale and enforced two key dynamic constraints using GA for the following qualitative fitting: (i) the peak amplitude of the secondary NFAT activation response is > 2-fold higher than its primary one^11^; and (ii) NFAT response is transient in both cases^49^. The cut-off amplification index of 2 was chosen to ensure the fitted models have robust NFAT amplification. Through this process, we repeated this process to obtain 100 ensemble models that generate target features (Fig. 7a).

## Supplementary information

Supplementary Information for the manuscript

SBML model file of the CN-NFAT model
