# Supplementary material for: Coupled feedback regulation of nuclear factor of activated T-cells (NFAT) modulates activation-induced cell death of T cells
Source: Sci Rep. 2019 Jul 23;9:10637. doi: 10.1038/s41598-019-46592-z (PMC6650396; doi:10.1038/s41598-019-46592-z)
Supplement: Supplementary file 1 — Supplementary Information for the manuscript [file 41598_2019_46592_MOESM1_ESM.docx]

**Supplementary Information**

**for the manuscript**

**Coupled feedback regulation of nuclear factor of activated T-cells (NFAT) modulates activation-induced cell death of T cells**

Sung-Young Shin^1,2^, Min-Wook Kim^3^, Kwang-Hyun Cho^3,4,#^ and Lan K. Nguyen1,2,[[1]](#footnote-1)*

^1^ Department of Biochemistry and Molecular Biology, School of Biomedical Sciences, Monash University, Clayton, Victoria 3800, Australia.
^2^ Biomedicine Discovery Institute, Monash University, Clayton, Victoria 3800, Australia.

^3^ Graduate School of Medical Science and Engineering, Korea Advanced Institute of Science and Technology (KAIST) 291 Daehak-ro, Yuseong-gu, Daejeon 34141, Republic of Korea.

^4^ Department of Bio and Brain Engineering, Korea Advanced Institute of Science and Technology (KAIST) 291 Daehak-ro, Yuseong-gu, Daejeon 34141, Republic of Korea.

## **S1. Model training and numerical simulations**

Model training (or calibration) is a process that aims to fit a computational model to known and quantified experiment data whereby unmeasured model parameters (including kinetic and state variables) are estimated so that model simulations can recapitulate the data. Essentially, parameter estimation is an optimisation problem in which a carefully formulated objective function representing the discrepancy between model simulations and experimental data is minimised.

*Objective function:*

In this study, the following metric function (also called ‘cost function’) was used to quantify the discrepancy between simulated values and corresponding experimental measurements [1-3] :

$$J(p)=\sum_{j=1}^{M} \sum_{i=1}^{N} \left(\frac{y_{j,i}^{D}-y_{j}(t_{i},p)}{\sigma_{j,i}}\right)^{2}$$

where $M$is the number of the given experimental data sets used for fitting and $N$is the number of time points within each experimental data set. $y_{j}(t_{i},p)$ represents the numerical solution for the model state variable $y_{j}$evaluated at time $t_{i}$ and parameter set $p$; while$y_{j,i}^{D}$ is the mean value of the corresponding data point at $t_{i}$ with the associated error variance$\sigma_{j,i}$. For normally distributed measurement errors, this metric function corresponds to a Maximum Likelihood estimation [1].

*Genetic Algorithm for optimizing the objective function*

In this study, a Genetic Algorithm (GA) was used for parameter estimation [2,3] since it is particularly suited for optimization problems [2,4,5]. Genetic algorithms is a class of optimisation algorithm inspired by the process of natural selection central to biological evolution [2,3,5,6]. As such, GA repeatedly searches for and updates a population of candidate solutions (called individuals or phenotypes). Usually, the initial population is randomly generated, covering the entire range of possible solutions (called search space). The population size depends on the nature of the problem, but typically contains several hundreds to thousands of possible solutions. At each step, GA selects individuals from the existing population to breed a new generation. During successive generations, the population "evolves" toward an optimal solution. Sometimes, GA may have a tendency to converge toward local minima. In this case, we can repeat the GA process by increasing the population size and/or changing the mutation and crossover rates. GA is equipped with three main biologically-inspired rules to generate the next generation of parameter values from the current population: selection, crossover and mutation.

For this work, we implemented GA using the Global Optimization Toolbox and the function *ga* in MATLAB ® (The MathWorks. Inc. 2017b). Selection rules select the individual solutions with the best fitness values (called ‘elite solution’) from the current population. The elite count was set to 5% of the population size. Crossover rules combine two parents to generate offspring for the next generation. The crossover fraction was set at 0.8. Note that for the setting of the elite rate and the crossover faction we used conventional values most commonly used in GA and also in our previous studies [2,3]. Mutation rules apply random changes to individual parents to generate the population of the next generation. For the mutation rule, we generated a random number from a Gaussian distribution with mean 0 and standard deviation σ_k_, which was applied to the individuals of the current generation. The standard deviation function (σ_k_) is given by the recursive formula as follows:

$\sigma_{k}=\sigma_{k-1}\left(1-\frac{k}{G}\right)$,

where *k* is the *k*^th^ generation, *G* is the number of generation, and $\sigma_{0}=1$.

In this study, we carried out repeated GA runs with a population size of 5,000 and generation number of 100. During this optimisation process, we also changed the mutation and crossover rates and even the population size to avoid the solution being trapped in local minima. After multiple repetitions of the GA process where the best-fitted parameters obtained from a previous repeat was used as the starting point of the next repeat, we arrived at the final best-fitted set when the objective function was no further reduced, and thus the optimal parameter values no longer changed. This quantitative optimisation procedure thus provided us confidence that the used parameter set represents an optimal set which minimises the difference between simulations and data.

*Numerical implementation*

The model training procedures and all numerical simulations in this study were implemented using MATLAB. The computationally intensive model training was performed on a dedicated High Performance Computing (HPC) cluster available to our group at Monash University (<http://www.monash.edu>). This HPC facility consists of two Haswell CPU sockets with a total of 16 physical cores (or 32 hyperthreaded cores) at 3.20 GHz and 300 TB usable storage. Less intensive, e.g. time-course simulations, were performed using Matlab on a desktop PC (3.4 GHz quad core Intel i7-6700).

The Ordinary differential equations (ODEs) were integrated and solved using the function *ode15s* in Matlab, which is a variable-step and variable-order solver, based on the numerical differentiation formulas (NDFs) and is specially designed for stiff systems.

## **S2. Mathematical Model Description**

**Description of a mathematical model for the TCR-CN-NFAT network**

#### Activation of T cell receptor and the TCR-CN-NFAT pathway

TCR activation is initiated by the recognition of cognate peptide–major histocompatibility complex (MHC) molecules of antigen presenting cells (APCs) [7] (reaction 1, Fig. 1e). The engagement of the TCR with an agonist-peptide-MHC complex recruits the SRC family kinase member LCK to the TCR-CD3 complex (CD3 is a T-cell co-receptor that help to activate T cells), which phosphorylates the immunoreceptor tyrosine-based activation motifs (ITAM) of CD3 [7]. The phosphorylation of the ITAMs further recruits and activates ZAP70 by LCK. Activated ZAP70 then phosphorylates the LAT signalosome that includes phospholipase Cγ1 (PLCγ1) and growth factor receptor-bound protein 2 (GRB2). PLCγ1 activates cytosolic Ca^2+^ signaling pathway by catalyzing the hydrolysis of PIP3 to IP3 and DAG. On the other hand, GRB2 binding to the guanine nucleotide exchange factor SOS activate the ERK signaling pathway. As our model aims to capture the essential regulatory events rather than every biological details, we lumped the multiple TCR activation steps into simplified reactions where the MHC-engaged TCR/CD28 complex activates the CN-NFAT, ERK and PI3K pathways (reaction 1, 5, 52 and 59a). CD28 is a receptor protein that potentiates the TCR activation signal.

The TCR-CN-NFAT pathway plays a key role in linking TCR activation to NFAT-mediated transcription of genes associated with an immune response [8]. Upon TCR activation, elevated cytosolic Ca^2+^ binds to calmodulin (CaM) and the Ca^2+^/CaM complex binds to the regulatory domain of CN [9], triggering CN activation (reaction 5). CN then dephosphorylates multiple phosphoserines in the regulatory domain of NFAT, causing NFAT to translocate to the nucleus (reaction 10) where it cooperates with other transcriptional partners to initiate and maintain specific transcriptional programs [8]. In contrast, NFAT inactivation results from its being (re)phosphorylated by several kinases, in particular the serine/threonine protein kinase GSK3 (reaction 11), forcing NFAT to re-translocate to the cytoplasm [10,11].

#### RCAN-mediated negative and positive feedback loops

*RCAN* is a gene family that includes *RCAN1-3,* of which *RCAN1* is a specific NFAT’s transcriptional target (reaction 19) [12,13]. Interestingly, depending on its phosphorylation status, RCAN1 can differentially regulate CN activation. When unphosphorylated, RCAN inhibits CN through direct binding (reaction 7) [14,15] but TAK1-induced phosphorylations of RCAN1 at Ser94 and Ser136 switch its role from being an inhibitor to an activator of CN (reaction 8) [16]. As a result, RCAN mediates coupled positive and negative feedback loops towards NFAT [17], described by reactions 19, 21 and 23.

#### IL-2 mediated positive feedback loop

Interleukin-2 (IL-2) is a pleiotropic cytokine produced by helper T cells after antigenic stimulation that is critical in mediating immune responses [18]. TCR stimulation and CN-induced NFAT activation lead to transcription of the IL-2 receptor (IL-2R) and secretion of IL-2 (reaction 43), essential for T cell proliferation during the clonal expansion phase. Importantly, secreted IL-2 activates IL-2R (reaction 45), leading to activation of the PI3K/Akt pathway as a major effector pathway [19] (reaction 59b). Activated Akt phosphorylates and inhibits GSK3 (reaction 65). This closes a positive feedback loop between IL-2/IL-2R, PI3K/Akt/GSK3 and NFAT that may promote NFAT activity amplification.

#### NFAT auto-regulatory positive feedback loop

The NFAT protein family consists of five members NFAT1-5. NFAT1-4 are regulated by calcium signaling while NFAT5 is regulated by osmotic stress [11]. NFAT2 and 3 expressions are strongly induced following TCR stimulation and maintained by positive auto-regulation [20-22]. NFAT1 and 4 also form an auto-amplification feedback loop mediated through miRNAs [23,24]. For simplicity, we do not consider separate isoforms of NFAT in our model but instead model NFAT as a single network entity, and describe its positive feedback loop by reactions 13 and 15.

*Carabin-mediated negative feedback loops*

Carabin is known as an endogenous inhibitor of CN through direct inhibitory binding [25]. Previous studies have also revealed that the Carabin gene has multiple consensus NFAT-binding sites and the expression of Carabin is regulated by the CN signaling pathway [26]. These observations indicate that Carabin negatively feeds back to CN (reactions 9 and 26). Moreover, Carabin has demonstrated GAP activity towards Ras that suppresses ERK signalling during TCR signaling [26]. Thus, Carabin forms an additional negative feedback mechanism through Ras, described by reactions 26 and 54.

*CTLA-4-mediated negative feedback loop*

Cytotoxic T-lymphocyte-associated protein 4 (CTLA-4) is a cell-surface molecule expressed nearly exclusively in CD4+ and CD8+ T cells [27], and known to be a negative regulator of TCR activation through dephosphorylation of ZAP70 (a component of the TCR complex) (reaction 2). Moreover, CTLA-4 suppresses PI3K/Akt signalling by enhancing PP2A-induced dephosphorylation of Akt [28] (reaction 61). The CTLA-4 gene has a consensus NFAT-binding sequence and binds NFAT with high affinity [29]. Furthermore, CTLA-4 was preferentially expressed in activated CD4+ T cells [27]. Together, these data suggest that CTLA-4 mediates a negative feedback loop towards the TCR-CN-NFAT axis (reactions 38, 40, 2).

**Supplementary Table S1. Reactions and reaction rates of the TCR-CN-NFAT signaling network model (model scheme given in Fig.1e, main text).**

| **No** | **Reaction** | **Reaction rates** |
| --- | --- | --- |
| re_1 | TCR → pTCR | kc1*E1*TCR/(km1+TCR) |
| re_2 | pTCR → TCR | kc2*(CTLA4)*pTCR/(km2+pTCR) |
| re_3 | pTCR → iTCR | v3*pTCR/(km3+pTCR) |
| re_4 | iTCR → TCR | v4*iTCR/(km4+iTCR) |
| re_5 | CN → aCN | kc5*pTCR*CN/(km5+CN) |
| re_6 | aCN → CN | v6*aCN/(km6+aCN) |
| re_7 | aCN+RCAN ↔ aCNRCAN | ka7*aCN*RCAN - kd7*aCNRCAN |
| re_8 | aCN+pRCAN ↔ aCNpRCAN | ka8*aCN*pRCAN - kd8*aCNpRCAN |
| re_9 | aCN+Carabin ↔ aCNCarabin | ka9*aCN*Carabin - kd9*aCNCarabin |
| re_10 | pNFAT → NFAT | kc10*(aCN+aCNpRCAN)*pNFAT/(km10+pNFAT) |
| re_11 | NFAT → pNFAT | (v11+kc11*GSK3)*NFAT/(km11+NFAT) |
| re_12 | ∅ → mNFAT | vs12 |
| re_13 | ∅ → mNFAT | v12*NFAT/(km12+NFAT) |
| re_14 | mNFAT → ∅ | kdeg12*mNFAT |
| re_15 | ∅ → NFAT | v13*mNFAT/(km13+mNFAT) |
| re_16 | NFAT → ∅ | kdeg13*NFAT |
| re_17 | pNFAT → ∅ | kdeg13*pNFAT |
| re_18 | ∅ → mRCAN | vs14 |
| re_19 | ∅ → mRCAN | v14*NFAT^2/(km14^2+NFAT^2) |
| re_20 | mRCAN → ∅ | kdeg14*mRCAN |
| re_21 | ∅ → RCAN | v15*mRCAN/(km15+mRCAN) |
| re_22 | RCAN → ∅ | kdeg15*RCAN |
| re_23 | RCAN → pRCAN | kc16*pTAK1*RCAN/(km16+RCAN) |
| re_24 | pRCAN → RCAN | v17*pRCAN/(km17+pRCAN) |
| re_25 | ∅ → mCarabin | vs18 |
| re_26 | ∅ → mCarabin | v18*NFAT^2/(km18^2+NFAT^2) |
| re_27 | mCarabin → ∅ | kdeg18*mCarabin |
| re_28 | ∅ → Carabin | v19*mCarabin/(km19+mCarabin) |
| re_29 | Carabin → ∅ | kdeg19*Carabin |
| re_30 | ∅ → mTNFa | vs20 |
| re_31 | ∅ → mTNFa | v20*NFAT^2/(km20^2+NFAT^2) |
| re_32 | mTNFa → ∅ | kdeg20*mTNFa |
| re_33 | ∅ → TNFa | v21*mTNFa/(km21+mTNFa) |
| re_34 | TNFa → ∅ | kdeg21*TNFa |
| re_35 | TAK1 → pTAK1 | kc22*(TNFa)*TAK1/(km22+TAK1) |
| re_36 | pTAK1 → TAK1 | kc23*pTAK1/(km23+pTAK1) |
| re_37 | ∅ → mCTLA4 | vs24 |
| re_38 | ∅ → mCTLA4 | v24*NFAT^2/(km24^2+NFAT^2) |
| re_39 | mCTLA4 → ∅ | kdeg24*mCTLA4 |
| re_40 | ∅ → CTLA4 | v25*mCTLA4/(km25+mCTLA4) |
| re_41 | CTLA4 → ∅ | kdeg25*CTLA4 |
| re_42 | ∅ → mIL2 | vs26 |
| re_43 | ∅ → mIL2 | v26a*(1+v26b*pERK/(km26b+pERK))*NFAT^2/(km26^2+NFAT^2) |
| re_44 | mIL2 → ∅ | kdeg26*mIL2 |
| re_45 | ∅ → IL2 | v27*mIL2/(km27+mIL2) |
| re_46 | IL2 →∅ | kdeg27*IL2 |
| re_47 | ∅ → mFasL | vs28 |
| re_48 | ∅ → mFasL | v28a*(1+v28b*pERK/(km28b+pERK))*NFAT^5/(km28^5+NFAT^5) |
| re_49 | mFasL → ∅ | kdeg28*mFasL |
| re_50 | ∅ → FasL | v29*mFasL/(km29+mFasL) |
| re_51 | FasL → ∅ | kdeg29*FasL |
| re_52 | Ras → aRas | kc30*pTCR*Ras/(km30+Ras)/(1+pERK/ki30) |
| re_53 | aRas → Ras | v31*aRas/(km31+aRas) |
| re_54 | Carabin+aRas ↔ aRasCarabin | ka32*aRas*Carabin - kd32*aRasCarabin |
| re_55 | MEK → pMEK | (kc33a*aRas+kc33b*Rac1GTP)*MEK/(km33+MEK) |
| re_56 | pMEK → MEK | v34*pMEK/(km34+pMEK) |
| re_57 | ERK → pERK | kc35*pMEK*ERK/(km35+ERK) |
| re_58 | pERK → ERK | v36*pERK/(km36+pERK) |
| re_59 | PI3K → aPI3K | (kc37a*pIL2R+kc37b*pTCR)*PI3K/(km37+PI3K) |
| re_60 | aPI3K → PI3K | v38*aPI3K/(km38+aPI3K) |
| re_61 | PP2A → aPP2A | kc39*CTLA4*PP2A/(km39+PP2A) |
| re_62 | aPP2A → PP2A | v40*aPP2A/(km40+aPP2A) |
| re_63 | Akt → pAkt | kc41*aPI3K*Akt/(km1+Akt) |
| re_64 | pAkt → Akt | kc42*aPP2A*pAkt/(km42+pAkt) |
| re_65 | GSK3 → pGSK3 | kc43*pAkt*GSK3/(km43+GSK3) |
| re_66 | pGSK3 → GSK3 | vs44*pGSK3/(km44+pGSK3) |
| re_67 | Rac1GDP → Rac1GTP | kc45*aPI3K*Rac1GDP/(km45+Rac1GDP) |
| re_68 | Rac1GTP → Rac1GDP | v46*Rac1GTP/(km46+Rac1GTP) |
| re_69 | aCN → aCNCnI | ka47*aCN*CnI - kd47*aCNCnI |
| re_70 | IL2R → pIL2R | kc48*IL2*IL2R/(km48+IL2R) |
| re_71 | pIL2R → IL2R | kc49*pIL2R/(km49+pIL2R) |
| re_72 | pIL2R → iIL2R | v50*pIL2R/(km50+pIL2R) |
| re_73 | iIL2R → pIL2R | v51*iIL2R/(km51+iIL2R) |

E1 represents the TCR stimulation as an input of the model. The effects of the CN inhibitor was modelled according to to a reversible drug-target binding reaction (as in v74) since CnI was reported to selectively inhibit dimerization and activation of calcineurin [30,31]. We assumed that the STAT3 inhibitor directly bind and form an inhibition complex and ka74 and kd74 denotes the binding coefficients. Note that a simple arrow (→) indicates the reaction is a catalytic reaction, while a double-sided arrow (↔) indicates the reaction combines an association and a dissociation process. The symbol ∅ with an arrow at the beginning (→ ∅) indicates the reaction is a degradation event; and the symbol ∅ with an arrow at the end (∅ →) indicates the reaction is a synthesis (transcriptional and translational) event.

**Supplementary Table S2. Ordinary differential equations of the TCR-CN-NFAT network model (model scheme given in Fig.1e, main text).** The reaction rates are given in Supplementary Table S1.

| **Left-had Sides** | **Right-hand Sides** | **Initial Conditions (nM)** |
| --- | --- | --- |
| d[TCR]/dt | - re_1 + re_2 + re_4 | 1.000E+01 |
| d[pTCR]/dt | + re_1 - re_2 - re_3 | 4.384E-22 |
| d[iTCR]/dt | + re_3 - re_4 | 1.937E-23 |
| d[CN]/dt | - re_5 + re_6 | 1.000E+01 |
| d[aCN]/dt | + re_5 - re_6 - re_7 - re_8 - re_9 - re_69 | 2.262E-18 |
| d[aCNRCAN]/dt | + re_7 | 2.209E-20 |
| d[aCNpRCAN]/dt | + re_8 | 2.416E-19 |
| d[aCNCarabin]/dt | + re_9 | 4.286E-17 |
| d[NFAT]/dt | + re_10 - re_11 + re_15 - re_16 | 1.283E-03 |
| d[pNFAT]/dt | - re_10 + re_11 - re_17 | 9.906E+00 |
| d[mNFAT]/dt | + re_12 + re_13 - re_14 | 1.955E-01 |
| d[mRCAN]/dt | + re_18 + re_19 - re_20 | 1.000E-01 |
| d[RCAN]/dt | - re_7 + re_21 - re_22 - re_23 + re_24 | 1.000E-01 |
| d[pRCAN]/dt | - re_8 + re_23 - re_24 | 1.290E-02 |
| d[mCarabin]/dt | + re_25 + re_26 - re_27 | 1.000E-01 |
| d[Carabin]/dt | - re_9 + re_28 - re_29 - re_54 | 1.000E-01 |
| d[mTNFa]/dt | + re_30 + re_31 - re_32 | 1.200E-01 |
| d[TNFa]/dt | + re_33 - re_34 | 1.200E-01 |
| d[TAK1]/dt | - re_35 + re_36 | 9.239E+00 |
| d[pTAK1]/dt | + re_35 - re_36 | 7.611E-01 |
| d[mCTLA4]/dt | + re_37 + re_38 - re_39 | 1.000E-01 |
| d[CTLA4]/dt | + re_40 - re_41 | 1.000E-01 |
| d[mIL2]/dt | + re_42 + re_43 - re_44 | 1.000E-01 |
| d[IL2]/dt | + re_45 - re_46 | 1.000E-01 |
| d[mFasL]/dt | + re_47 + re_48 - re_49 | 1.000E-01 |
| d[FasL]/dt | + re_50 - re_51 | 1.000E-01 |
| d[Ras]/dt | - re_52 + re_53 | 1.000E+01 |
| d[aRas]/dt | + re_52 - re_53 - re_54 | 8.375E-25 |
| d[aRasCarabin]/dt | + re_54 | 8.472E-31 |
| d[MEK]/dt | - re_55 + re_56 | 9.999E+00 |
| d[pMEK]/dt | + re_55 - re_56 | 9.077E-04 |
| d[ERK]/dt | - re_57 + re_58 | 1.000E+01 |
| d[pERK]/dt | + re_57 - re_58 | 4.769E-04 |
| d[PI3K]/dt | - re_59 + re_60 | 1.000E+01 |
| d[aPI3K]/dt | + re_59 - re_60 | 5.945E-05 |
| d[PP2A]/dt | - re_61 + re_62 | 9.996E+00 |
| d[aPP2A]/dt | + re_61 - re_62 | 3.959E-03 |
| d[Akt]/dt | - re_63 + re_64 | 8.853E+00 |
| d[pAkt]/dt | + re_63 - re_64 | 1.147E+00 |
| d[GSK3]/dt | - re_65 + re_66 | 4.507E+00 |
| d[pGSK3]/dt | + re_65 - re_66 | 5.493E+00 |
| d[Rac1GDP]/dt | - re_67 + re_68 | 9.892E+00 |
| d[Rac1GTP]/dt | + re_67 - re_68 | 1.078E-01 |
| d[aCNCnI]/dt | + re_69 | 0.000E+00 |
| d[IL2R]/dt | - re_70 + re_71 | 5.258E-01 |
| d[pIL2R]/dt | + re_70 - re_71 - re_72 + re_73 | 9.442E+00 |
| d[iIL2R]/dt | + re_72 - re_73 | 3.187E-02 |

**Supplementary Table S3. Best-fitted parameter values used for simulations.**

| **Parameter** | **Value** | **Unit** | **Parameter** | **Value** | **Unit** |
| --- | --- | --- | --- | --- | --- |
| kc1 | 3.289E+01 | min^-1^ | v20 | 8.974E-02 | nM min^-1^ |
| km1 | 1.483E-01 | nM | km20 | 3.428E-02 | nM |
| kc2 | 1.200E+01 | min^-1^ | kdeg20 | 6.281E-03 | min^-1^ |
| km2 | 1.138E+01 | nM | km43 | 2.655E+01 | nM |
| v3 | 1.161E+02 | nM min^-1^ | km21 | 5.998E+04 | nM |
| km3 | 1.000E+05 | nM | kdeg21 | 6.012E-02 | min^-1^ |
| v4 | 6.353E-01 | nM min^-1^ | kc22 | 2.018E+01 | min^-1^ |
| km4 | 2.911E+03 | nM | km22 | 3.890E+01 | nM |
| kc5 | 6.012E+04 | min^-1^ | kc23 | 1.545E+01 | min^-1^ |
| km5 | 1.282E+01 | nM | km23 | 2.455E+01 | nM |
| v6 | 3.999E+00 | nM min^-1^ | kc43 | 1.021E+04 | min^-1^ |
| km6 | 9.036E+01 | nM | v24 | 7.079E+00 | nM min^-1^ |
| ka7 | 9.247E-02 | nM^-1^ min^-1^ | km24 | 3.097E+01 | nM |
| kd7 | 2.138E+00 | min^-1^ | kdeg24 | 1.028E+01 | min^-1^ |
| ka8 | 2.582E+03 | nM^-1^ min^-1^ | km40 | 4.955E-02 | nM |
| kd8 | 3.155E+02 | min^-1^ | km25 | 5.861E+02 | nM |
| ka9 | 1.455E+00 | nM^-1^ min^-1^ | kdeg25 | 3.034E+04 | min^-1^ |
| kd9 | 1.219E-01 | min^-1^ | km42 | 4.325E+03 | nM |
| kc10 | 2.767E+02 | min^-1^ | v26a | 4.159E+04 | nM min^-1^ |
| km10 | 1.871E+03 | nM | v26b | 2.500E-01 | nM min^-1^ |
| v11 | 4.977E+01 | nM min^-1^ | km26 | 3.048E+02 | nM |
| kc11 | 4.477E+01 | min^-1^ | kdeg26 | 8.831E-02 | min^-1^ |
| km11 | 2.317E+01 | nM | kc42 | 1.119E+04 | min^-1^ |
| km46 | 8.590E+03 | nM | km27 | 3.784E+01 | nM |
| v12 | 9.594E+01 | nM min^-1^ | kdeg27 | 2.979E-03 | min^-1^ |
| km12 | 4.943E-02 | nM | km41 | 2.323E+02 | nM |
| kdeg12 | 2.541E+01 | min^-1^ | v28a | 3.027E+02 | nM min^-1^ |
| v13 | 1.791E+00 | nM min^-1^ | v28b | 2.500E-01 | nM min^-1^ |
| km13 | 2.495E+01 | nM | v40 | 5.140E+00 | nM min^-1^ |
| kdeg13 | 1.403E-03 | min^-1^ | kdeg28 | 6.427E-03 | min^-1^ |
| v46 | 1.352E+02 | nM min^-1^ | kc41 | 5.383E+03 | min^-1^ |
| v14 | 4.819E+00 | nM min^-1^ | km29 | 4.055E+04 | nM |
| km14 | 5.433E+00 | nM | kdeg29 | 1.858E+00 | min^-1^ |
| kdeg14 | 1.361E+00 | min^-1^ | kc30 | 5.370E+00 | min^-1^ |
| km45 | 2.099E+02 | nM | km30 | 1.130E+02 | nM |
| km15 | 2.113E+04 | nM | ki30 | 2.704E+03 | nM |
| kdeg15 | 8.279E+01 | min^-1^ | v31 | 5.458E+01 | nM min^-1^ |
| kc16 | 5.047E+03 | min^-1^ | km31 | 2.388E-01 | nM |
| km16 | 5.445E+04 | nM | ka32 | 9.376E-01 | nM^-1^ min^-1^ |
| v17 | 5.272E+01 | nM min^-1^ | kd32 | 9.268E+04 | min^-1^ |
| km17 | 9.638E+01 | nM | kc33a | 1.227E+04 | min^-1^ |
| kc45 | 6.339E+02 | min^-1^ | kc33b | 2.518E+01 | min^-1^ |
| v18 | 6.714E-02 | nM min^-1^ | km33 | 9.057E+02 | nM |
| km18 | 3.656E+00 | nM | v34 | 4.539E+01 | nM min^-1^ |
| kdeg18 | 1.905E-02 | min^-1^ | km34 | 1.390E+00 | nM |
| km44 | 6.095E-01 | nM | kc35 | 3.327E+04 | min^-1^ |
| km19 | 9.863E+01 | nM | km35 | 3.258E+01 | nM |
| kdeg19 | 2.173E+02 | min^-1^ | v36 | 1.750E+03 | nM min^-1^ |
| vs44 | 1.888E+03 | nM min^-1^ | km36 | 1.172E-01 | nM |
| kc37a | 1.982E-03 | min^-1^ | km26b | 2.449E-03 | nM |
| kc37b | 7.925E+02 | min^-1^ | km28b | 7.015E+02 | nM |
| km37 | 1.107E+03 | nM | km28 | 1.052E+02 | nM |
| v38 | 3.319E+01 | nM min^-1^ | vs12 | 2.541E+00 | nM min^-1^ |
| km38 | 1.178E+01 | nM | vs14 | 1.361E-01 | nM min^-1^ |
| kc39 | 1.442E+02 | min^-1^ | v15 | 1.750E+06 | nM min^-1^ |
| km39 | 3.690E+02 | nM | vs18 | 1.905E-03 | nM min^-1^ |
| ka47 | 2.042E+02 | nM^-1^ min^-1^ | v19 | 2.145E+04 | nM min^-1^ |
| kd47 | 1.486E+00 | min^-1^ | vs20 | 6.281E-04 | nM min^-1^ |
| kc48 | 1.486E+00 | min^-1^ | v21 | 3.606E+03 | nM min^-1^ |
| km48 | 1.486E+00 | nM | vs24 | 1.028E+00 | nM min^-1^ |
| kc49 | 9.226E+00 | min^-1^ | v25 | 1.779E+07 | nM min^-1^ |
| km49 | 2.234E+03 | nM | vs26 | 8.831E-03 | nM min^-1^ |
| v50 | 2.606E+01 | nM min^-1^ | v27 | 1.130E-01 | nM min^-1^ |
| km50 | 8.730E+00 | nM | vs28 | 6.427E-04 | nM min^-1^ |
| v51 | 2.588E+01 | nM min^-1^ | v29 | 7.534E+04 | nM min^-1^ |
| km51 | 2.904E-02 | nM | stim | 1.000E+02 | - |
| ton | 0 | min | CnI | (changeable) |  |
| toff | 2 | min |  |  |  |

**Supplementary Table S4. Parameters perturbed for feedback functional analysis shown in Fig. 3b-e, Fig. 5f-g and Fig. 7d.**

|  | **Interaction** | **Parameter (reaction no)** |  | **Interaction** | **Parameter**  **(reaction no)** |
| --- | --- | --- | --- | --- | --- |
| **F1** | TNFalpah --> TAK1 | kc22 (re_35) | **F7** | CTLA-4 --> PP2A | kc39 (re_61) |
| **F2** | RCAN --| CN | ka7 (re_7) | **F8** | PI3k --> Akt | kc41 (re_63) |
| **F3** | pRCNA --> CN | ka8 (re_8) | **F9** | Akt --> pGSK3 | kc43 (re_65) |
| **F4** | Carabin --| CN | ka9 (re_9) | **F10** | PI3K --> Rac1 | kc45 (re_67) |
| **F5** | Carabin --| Ras | ka32 (re_54) | **F11** | NFAT --> NFAT | re_13 (re_15) |
| **F6** | CTLA4 --| pTCR | kc2 (re_2) |  |  |  |

**Supplementary Table S5. Reactions and reaction rates of** **the reduced network model (model scheme given in Fig.6a, main text).**

|  | **Reaction** | **Reaction rates** |
| --- | --- | --- |
| v1 | TCR → pTCR | kc1* MHC*TCR/(1+Ki1*CTLA4) |
| v2 | pTCR → TCR | Vmax2*pTCR |
| v3 | CN → aCN | kc3* pTCR*CN |
| v4 | aCN → CN | Vmax4*aCN |
| v5 | pNFAT → NFAT | kc5a*aCN*(1+kc5b*pRCAN)*pNFAT/(1+Ki5*CARABIN) |
| v6 | NFAT → pNFAT | Vmax6*NFAT |
| v7 | ∅→ NFAT | kc7*NFAT/(Km7+NFAT) |
| v8 | NFAT →∅ | kdeg8*NFAT |
| v9 | ∅→ pRCAN | kc9*NFAT/(Km9+NFAT) |
| v10 | pRCAN →∅ | kdeg10*pRCAN |
| v11 | ∅→ CARABIN | kc11*NFAT/(Km11+NFAT) |
| V12 | CARABIN →∅ | kdeg12*pRCAN |
| V13 | ∅→ CTLA4 | kc13*NFAT/(Km13+NFAT) |
| V14 | CTLA4 →∅ | kdeg14*CTLA4 |

**Supplementary Table S6. Ordinary differential equations of the TCR-CN-NFAT network model (model scheme given in Fig.6a, main text).** The reaction rates are given in Supplementary Table S5.

| **Left-had Sides** | **Right-hand Sides** | **Initial Conditions (nM)** |
| --- | --- | --- |
| d[aCN]/dt | v3-v4 | 0 |
| d[pNFAT]/dt | -v5+v6 | 0 |
| d[pTCR]/dt | v1-v2 | 0 |
| d[TCR]/dt | -v1+v2 | 100 |
| d[CN]/dt | -v3+v4 | 100 |
| d[NFAT]/dt | v5-v6+v7-v8 | 30 |
| d[pRCAN]/dt | v9-v10 | 10 |
| d[CARABIN]/dt | v11-v12 | 10 |
| d[CTLA4]/dt | v13-v14 | 10 |

## **S3. Supplementary Figures**


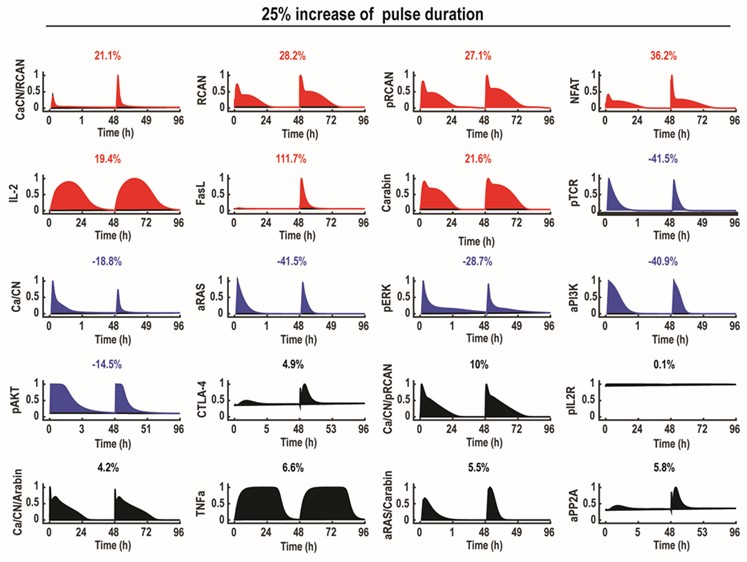


**Supplementary Figure S1**. **Network-wide responses to sequential (primary followed by secondary) TCR stimulations (****25% increase of pulse duration).** See also Fig. 2a in the main text.


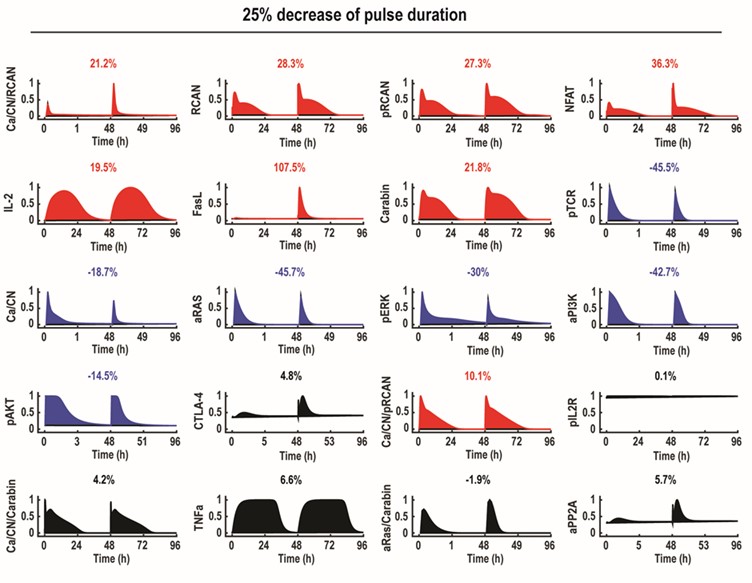


**Supplementary Figure S2**. **Network-wide responses to sequential (primary followed by secondary) TCR stimulations (****25% decrease of pulse duration).** See also Fig. 2a in the main text.


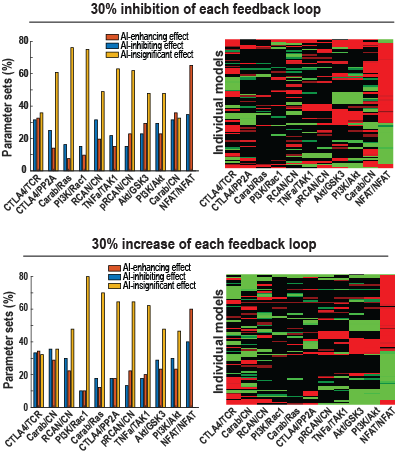


**Supplementary Figure S3**. **Tabulation of individual models where each feedback displays an AI-enhancing (red) or AI-inhibiting effect (blue) (left panels).** **Heatmap showing the color-coded sensitivity scores from a sensitivity analysis of various model feedback mechanisms on NFAT amplification (right panels).** Each feedback loops were perturbed by +/-30% for sensitivity analyses. See also Fig 7d in the main text.

**
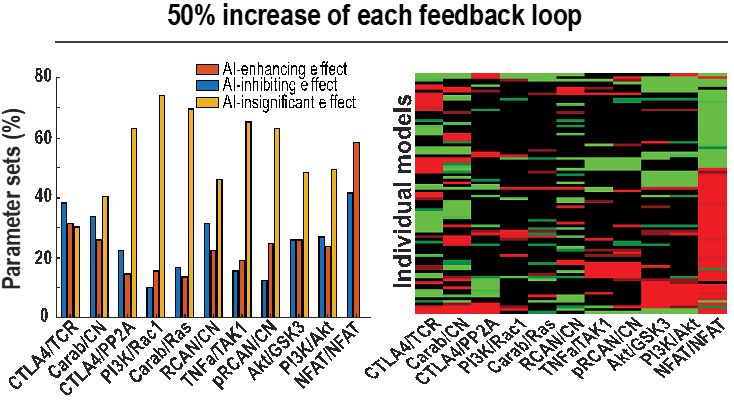
**

**Supplementary Figure S4**. **Tabulation of individual models where each feedback displays an AI-enhancing (red) or AI-inhibiting effect (blue) (left panels).** **Heatmap showing the color-coded sensitivity scores from a sensitivity analysis of various model feedback mechanisms on NFAT amplification (right panels).** Each feedback loops were perturbed by +50% for sensitivity analyses. See also Fig 7d in the main text.

**
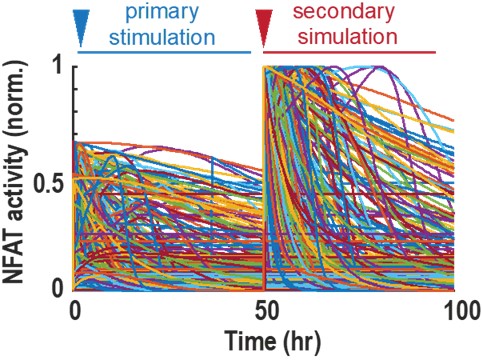
**

**Supplementary Figure S5**. Simulated time profiles showing NFAT activation amplification to a sequential TCR stimulation by the reduced model for different randomly sampled parameter sets (see also Fig 6 in the main text).

**
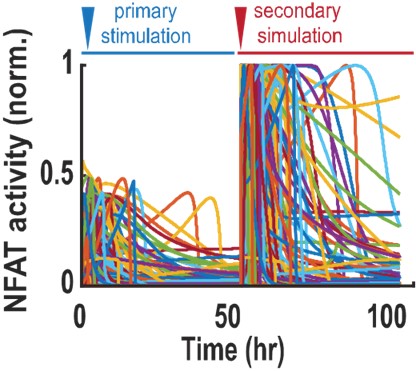
**

**Supplementary Figure S6**. Simulated time profiles of NFAT activation, superimposed for all the individual models (see also Fig 7 in the main text).

**Supplementary References**

1. Raue, A.; Kreutz, C.; Maiwald, T.; Bachmann, J.; Schilling, M.; Klingmüller, U.; Timmer, J. Structural and practical identifiability analysis of partially observed dynamical models by exploiting the profile likelihood. *Bioinformatics* **2009**, *25*, 1923-1929.

2. Shin, S.Y.; Kim, T.; Lee, H.S.; Kang, J.H.; Lee, J.Y.; Cho, K.H.; Kim do, H. The switching role of b-adrenergic receptor signalling in cell survival or death decision of cardiomyocytes. *Nat Commun* **2014**, *5*, 5777.

3. Shin, D.; Kim, I.S.; Lee, J.M.; Shin, S.Y.; Lee, J.H.; Baek, S.H.; Cho, K.H. The hidden switches underlying roralpha-mediated circuits that critically regulate uncontrolled cell proliferation. *J Mol Cell Biol* **2014**, *6*, 338-348.

4. Reali, F.; Priami, C.; Marchetti, L. Optimization algorithms for computational systems biology. *Frontiers in Applied Mathematics and Statistics* **2017**, *3*.

5. Man, K.F.; Tang, K.S.; Kwong, S. Genetic algorithms: Concepts and applications [in engineering design]. *IEEE Transactions on Industrial Electronics* **1996**, *43*, 519-534.

6. Srinivas, M.; Patnaik, L.M. Genetic algorithms: A survey. *Computer* **1994**, *27*, 17-26.

7. Brownlie, R.J.; Zamoyska, R. T cell receptor signalling networks: Branched, diversified and bounded. *Nat Rev Immunol* **2013**, *13*, 257-269.

8. Müller, M.R.; Rao, A. Nfat, immunity and cancer: A transcription factor comes of age. *Nat Rev Immunol* **2010**, *10*, 645-656.

9. Rumi-Masante, J.; Rusinga, F.I.; Lester, T.E.; Dunlap, T.B.; Williams, T.D.; Dunker, A.K.; Weis, D.D.; Creamer, T.P. Structural basis for activation of calcineurin by calmodulin. *Journal of Molecular Biology* **2012**, *415*, 307-317.

10. Hogan, P.G.; Chen, L.; Nardone, J.; Rao, A. Transcriptional regulation by calcium, calcineurin, and nfat. *Genes & development* **2003**, *17*, 2205-2232.

11. Macian, F. Nfat proteins: Key regulators of t-cell development and function. *Nat Rev Immunol* **2005**, *5*, 472-484.

12. Harris, C.D.; Ermak, G.; Davies, K.J.A. Multiple roles of the dscr1 (adapt78 or rcan1) gene and its protein product calcipressin 1 (or rcan1) in disease. *Cellular and Molecular Life Sciences CMLS* **2005**, *62*, 2477-2486.

13. Chan, B.; Greenan, G.; McKeon, F.; Ellenberger, T. Identification of a peptide fragment of dscr1 that competitively inhibits calcineurin activity in vitro and in vivo. *Proc Natl Acad Sci U S A* **2005**, *102*, 13075-13080.

14. Rothermel, B.A.; McKinsey, T.A.; Vega, R.B.; Nicol, R.L.; Mammen, P.; Yang, J.; Antos, C.L.; Shelton, J.M.; Bassel-Duby, R.; Olson, E.N.*, et al.* Myocyte-enriched calcineurin-interacting protein, mcip1, inhibits cardiac hypertrophy in vivo. *Proc Natl Acad Sci U S A* **2001**, *98*, 3328-3333.

15. Vega, R.B.; Yang, J.; Rothermel, B.A.; Bassel-Duby, R.; Williams, R.S. Multiple domains of mcip1 contribute to inhibition of calcineurin activity. *J Biol Chem* **2002**, *277*, 30401-30407.

16. Liu, Q.; Busby, J.C.; Molkentin, J.D. Interaction between tak1-tab1-tab2 and rcan1-calcineurin defines a signalling nodal control point. *Nat Cell Biol* **2009**, *11*, 154-161.

17. Minami, T. Calcineurin-nfat activation and dscr-1 auto-inhibitory loop: How is homoeostasis regulated? *Journal of biochemistry* **2014**, *155*, 217-226.

18. Liao, W.; Lin, J.X.; Leonard, W.J. Interleukin-2 at the crossroads of effector responses, tolerance, and immunotherapy. *Immunity* **2013**, *38*, 13-25.

19. Chow, C.-W.; Rincón, M.; Davis, R.J. Requirement for transcription factor nfat in interleukin-2 expression. *Molecular and cellular biology* **1999**, *19*, 2300-2307.

20. Serfling, E.; Chuvpilo, S.; Liu, J.; Hofer, T.; Palmetshofer, A. Nfatc1 autoregulation: A crucial step for cell-fate determination. *Trends Immunol* **2006**, *27*, 461-469.

21. Serfling, E.; Chuvpilo, S.; Liu, J.; Höfer, T.; Palmetshofer, A. Nfatc1 autoregulation: A crucial step for cell-fate determination. *Trends in Immunology* **2006**

*27*, 461-469.

22. Arron, J.R.; Winslow, M.M.; Polleri, A.; Chang, C.P.; Wu, H.; Gao, X.; Neilson, J.R.; Chen, L.; Heit, J.J.; Kim, S.K.*, et al.* Nfat dysregulation by increased dosage of dscr1 and dyrk1a on chromosome 21. *Nature* **2006**, *441*, 595-600.

23. da Costa Martins, P.A.; Salic, K.; Gladka, M.M.; Armand, A.S.; Leptidis, S.; el Azzouzi, H.; Hansen, A.; Coenen-de Roo, C.J.; Bierhuizen, M.F.; van der Nagel, R.*, et al.* Microrna-199b targets the nuclear kinase dyrk1a in an auto-amplification loop promoting calcineurin/nfat signalling. *Nat Cell Biol* **2010**, *12*, 1220-1227.

24. Kannambath, S. Micro-rna feedback loops modulating the calcineurin/nfat signaling pathway. *Non-coding RNA* **2016**, *2*.

25. Liu, J.O. Calmodulin-dependent phosphatase, kinases, and transcriptional corepressors involved in t-cell activation. *Immunological reviews* **2009**, *228*, 184-198.

26. Pan, F.; Sun, L.; Kardian, D.B.; Whartenby, K.A.; Pardoll, D.M.; Liu, J.O. Feedback inhibition of calcineurin and ras by a dual inhibitory protein carabin. *Nature* **2007**, *445*, 433-436.

27. Chan, D.V.; Gibson, H.M.; Aufiero, B.M.; Wilson, A.J.; Hafner, M.S.; Mi, Q.S.; Wong, H.K. Differential ctla-4 expression in human cd4+ versus cd8+ t cells is associated with increased nfat1 and inhibition of cd4+ proliferation. *Genes Immun* **2014**, *15*, 25-32.

28. Wohlfert, E.A.; Clark, R.B. ‘Vive la résistance!’ – the pi3k–akt pathway can determine target sensitivity to regulatory t cell suppression. *Trends in Immunology* **2007**, *28*, 154-160.

29. Gibson, H.M.; Hedgcock, C.J.; Aufiero, B.M.; Wilson, A.J.; Hafner, M.S.; Tsokos, G.C.; Wong, H.K. Induction of the ctla-4 gene in human lymphocytes is dependent on nfat binding the proximal promoter. *The Journal of Immunology* **2007**, *179*, 3831-3840.

30. Calne, R.Y.; Rolles, K.; White, D.J.; Thiru, S.; Evans, D.B.; McMaster, P.; Dunn, D.C.; Craddock, G.N.; Henderson, R.G.; Aziz, S.*, et al.* Cyclosporin a initially as the only immunosuppressant in 34 recipients of cadaveric organs: 32 kidneys, 2 pancreases, and 2 livers. *Lancet* **1979**, *2*, 1033-1036.

31. Borel, J.F.; Feurer, C.; Gubler, H.U.; Stahelin, H. Biological effects of cyclosporin a: A new antilymphocytic agent. *Agents and actions* **1976**, *6*, 468-475.

1. ^*^ Correspondence: lan.k.nguyen@monash.edu (LKN); Tel.: +61-3-9905-1298.

   **^#^** Co-correspondence: ckh@kaist.ac.kr (KHC); Tel: +82-42-350-4325. [↑](#footnote-ref-1)
